# The dual impact of ictal and interictal burden in migraine: an analysis from the ObserVational survey of the Epidemiology, tReatment, and Care Of MigrainE (OVERCOME) Japan second study

**DOI:** 10.1186/s10194-025-02079-z

**Published:** 2025-06-16

**Authors:** Tsubasa Takizawa, Daisuke Danno, Ryotaro Ishii, Shiho Suzuki, Moemi Miura, Yoshinori Tanizawa, Satoshi Osaga, Michio Okada, Chie Hashimoto, Mika Komori

**Affiliations:** 1 Department of Neurology, Keio University School of Medicine, Tokyo, Japan; 2 Headache Center and Department of Neurology, Tominaga Hospital, Osaka, Japan; 3 Department of Neurology, Kyoto Prefectural University of Medicine, Kyoto, Japan; 4 Department of Neurology, Dokkyo Medical University, Tochigi, Japan; 5 Social Survey Research Information Co., Ltd., Tokyo, Japan; 6 Japan Drug Development and Medical Affairs, Eli Lilly Japan K.K., 5-1-28, Isogamidori, Chuo-Ku, Kobe-Shi, Hyogo 651-0086 Japan

**Keywords:** Burden of disease, Family, Ictal burden, Interictal burden, Japan, Migraine disorders, Patient-reported outcome measures, Quality of life, Surveys and questionnaires

## Abstract

**Background:**

Migraine adversely affects many aspects of daily life. In addition to burdens during headaches (ictal period), burdens between headaches (interictal period) are increasingly recognized. In this analysis of the ObserVational survey of the Epidemiology, tReatment, and Care Of MigrainE in Japan (OVERCOME [Japan]) 2nd study, we evaluated the contribution of interictal burden to daily activities, quality of life, work, family, costs, and medical treatment.

**Methods:**

The OVERCOME (Japan) 2nd study was a cross-sectional, web-based survey of 19,590 adults in Japan with migraine conducted between June and August 2023. Questionnaires included Headache Impact Test-6 (HIT-6), Migraine Interictal Burden Scale-4 (MIBS-4), Migraine Disability Assessment (MIDAS), Allodynia Symptom Checklist-12 (ASC-12), Migraine-Specific Quality-of-Life Questionnaire (MSQ), Impact of Migraine on Partners and Adolescent Children Scale (IMPAC), Work Productivity and Activity Impairment Questionnaire-Migraine (WPAI-M), and Migraine Treatment Optimization Questionnaire-6 (mTOQ-6). Additional questions asked about costs, frequency of migraine concerns between headaches, and experience with medical treatment. Analyses were conducted on subgroups based on HIT-6 and MIBS-4 scores: Severe (HIT-6 ≥ 60, MIBS-4 ≥ 3; *n* = 6854), High Interictal Burden (HIT-6 < 60, MIBS-4 ≥ 3; *n* = 2368), High Ictal Burden (HIT-6 ≥ 60, MIBS-4 < 3; *n* = 4253), and Milder (HIT-6 < 60, MIBS-4 < 3; *n* = 6115).

**Results:**

Number of monthly headache days, total MIDAS score, and pain severity score were higher, and MSQ scores lower, in subgroups with higher HIT-6 (HIT-High) versus subgroups with lower HIT-6 (HIT-Low). Within both HIT-High and HIT-Low subgroups, those with higher MIBS-4 had higher MIDAS and lower MSQ. IMPAC grade, ASC-12 score, and WPAI-M absenteeism were higher in the High Interictal Burden subgroup versus the High Ictal Burden subgroup. Concerns between headaches were more frequent, and costs higher, in subgroups with higher MIBS-4 (MIBS-High) versus subgroups with lower MIBS-4 (MIBS-Low). Acute treatment prescription analgesics were more commonly used in HIT-High versus HIT-Low subgroups, but triptans, lasmiditan, and preventive drugs were more common in the High Interictal Burden subgroup versus the High Ictal Burden subgroup.

**Conclusions:**

These results revealed that high interictal burden negatively affects multiple aspects of daily life in Japanese people with migraine independently of the impact of headaches.

**Graphical abstract:**

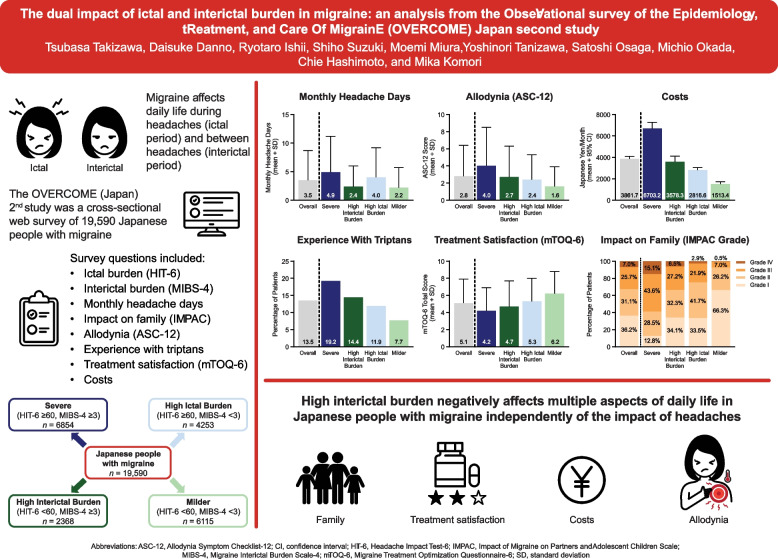

**Supplementary Information:**

The online version contains supplementary material available at 10.1186/s10194-025-02079-z.

## Introduction

Migraine is a neurological disorder that affects an estimated 1.16 billion people worldwide and is the second leading cause of years lived with disability among people aged 20–59 years [1]. In East Asia, the reported prevalence of migraine ranges from 6.0% to 14.3% among non-elderly adults (aged < 60 years) [2]. The primary symptom of migraine is moderate to severe headache, which is often accompanied by sensitivity to light or sound, nausea, dizziness, and cutaneous allodynia, all of which may occur before or during headache [3]. The pain and disability that accompany headaches adversely affect numerous aspects of daily life for people with migraine [3–5]. However, in addition to disability occurring during headaches (the ictal period), daily life is also greatly affected during the interictal period between headaches [3–5]. The interictal period may be characterized by psychological symptoms such as anxiety about the next migraine headache and its effects on work and family [4, 6, 7]. The physical symptoms, such as cutaneous allodynia, increased sensitivity to light and sound, and dizziness, that are commonly associated with headaches can also be present during the interictal period [3, 6, 7]. Although the burdens associated with the interictal period are increasingly recognized, additional research is needed to fully understand its impact on people with migraine.

Several instruments are routinely used to measure the effect of headaches on daily life in people with migraine, such as the Migraine Disability Assessment (MIDAS), a five-item questionnaire that measures headache-related disability [8], and the Headache Impact Test-6 (HIT-6), a six-item questionnaire that measures the impact of headaches on individual activities in work, school, home, and society [9, 10]. Although these questionnaires are among the most commonly used patient-reported outcome measures in headache disorders [11], they focus on the impact of effects occurring during headaches, i.e. ictal burden. To better understand interictal burden, Buse et al. developed a four-item questionnaire known as the Migraine Interictal Burden Scale-4 (MIBS-4) [12]. The MIBS-4 asks people how often headaches 1) affect work or school when they do not have a headache, 2) lead to worrying about planning social or leisure activities, 3) affect their lives when they do not have a headache, and 4) make them feel helpless between headaches. Each question is answered on a five-point Likert scale from “never” to “most of the time”, and total scores of 1–2, 3–4, and 5 + indicate mild, moderate, and severe interictal burden, respectively. The MIBS-4 is the only validated questionnaire to assess interictal burden and has been used in several observational studies [13–16] and at least one randomized trial [17], but more research is needed into the factors that contribute to or alleviate interictal burden.

In Japan, approximately 7.3%–8.4% of adults are affected by migraine [18, 19]. However, until recently, only a few studies had examined the humanistic or economic burden of migraine in Japan or other East Asian countries [2]. The ObserVational survey of the Epidemiology, tReatment, and Care Of MigrainE in Japan (OVERCOME [Japan]) study, a nationwide, web-based survey conducted in 2020, provided extensive information on the status of more than 17,000 people with migraine in Japan [20–25]. The OVERCOME (Japan) study established that migraine has a substantial impact on daily activities [20], quality of life [24], work [22, 24], and family [20] in Japanese adults. Substantial unmet needs in medical care were identified, with 36.5% of respondents hesitating to seek treatment for migraine and 89.8% never having used preventive drugs [24]. Almost half (41.5%) of respondents with migraine reported moderate to severe levels of interictal burden measured by MIBS-4 [24], which was directly related to poor satisfaction with treatment effectiveness [21]. However, the relative effects of interictal burden compared with ictal burden on the lives of people with migraine have not been assessed. In addition, the characteristics of people with high interictal burden have not been previously evaluated.

The landscape for migraine treatment in Japan has evolved rapidly in the years since OVERCOME (Japan) was completed in 2020, with the approval of new classes of drugs, including the selective 5-HT_1 F_ receptor agonist lasmiditan for acute treatment [26, 27] and monoclonal antibodies (mAbs) targeting calcitonin gene-related peptide (CGRP) for preventive treatment [28–31]. To assess how the availability of new treatment options has affected people with migraine in Japan, the OVERCOME (Japan) 2nd study was conducted in 2023. Results from the OVERCOME (Japan) 2nd study on the impact of migraine throughout the patient’s life, as well as a snapshot of current treatment patterns, are reported elsewhere [32]. In this analysis of the OVERCOME (Japan) 2nd study, we evaluated the relationship between the level of ictal/interictal burden and the impact of migraine on daily activities, quality of life, work, and family. In addition, we assessed how ictal and interictal burdens affect the costs associated with migraine and experiences with medical consultations and drug treatments.

## Methods

### Study design

The ObserVational survey of the Epidemiology, tReatment, and Care Of MigrainE (OVERCOME) Japan 2nd study was a cross-sectional, web-based survey study of people in Japan with and without migraine [32]. The study was conducted from June 14 to August 28, 2023, in accordance with ethical principles originating from the Declaration of Helsinki and consistent with Good Pharmacoepidemiology Practices and all applicable Japanese laws and regulations. The study protocol was approved by the Ethics Review Board of the Medical Corporation TOUKEIKAI Kitamachi Clinic (BGQ09531) on April 19, 2023. All survey participants provided informed consent.

### Study population

Participants aged ≥ 18 years and resident in Japan were recruited using survey panels from Cross Marketing Inc., GMO Research, Inc., and Rakuten Insight, Inc. (all based in Tokyo, Japan). Potential participants were sent an email containing a link to the online survey. Screening questions on sex, age, and geographic region were used to obtain a representative sample of Japan’s population based on 2020 census data [33]. Participants who met the screening criteria and provided informed consent could then proceed to the survey. Those who completed the survey received points from their respective survey panel as compensation; accumulated points could be redeemed for gift certificates.

On the basis of previous surveys, particularly the first OVERCOME (Japan) study [22], this study aimed to include approximately 20,000 participants with migraine. This target was based on the prevalence of migraine determined in the OVERCOME (Japan) study (8.6%) [22], the number of potential respondents in the survey panels, the response rate in previous surveys, and the feasibility of subgroup analyses. Participants were eligible for the migraine group if they had experienced headaches or migraine in the past 12 months, and either self-reported as having been diagnosed with migraine by a physician or were deemed to have migraine from responses to survey questions set in reference to modified International Classification of Headache Disorders, 3rd edition (ICHD-3) diagnostic criteria for migraine [34]. Inclusion in the migraine group was not limited by frequency of migraine headaches. Participants were excluded from the migraine group if all their headaches in the past 12 months were secondary headaches (caused by hangovers, illness, or trauma), if they reported having primary headaches but did not meet the modified ICHD-3 criteria, or if there were inconsistencies between the answers to sex and disease history questions. An additional group without migraine (target of approximately 2000 participants) was also recruited for analyses reported elsewhere [32].

### Outcome measures

Demographic data, including age, sex, marriage status, cohabitation status, employment status, and disease history, were collected from all participants. The migraine group also answered questions on their headaches (including their impact) and their treatment. Information about headaches during the past 3 months included the number of monthly headache days, the duration of headaches, and the frequency of allodynia symptoms, assessed using the Allodynia Symptom Checklist-12 (ASC-12) [35]. Information on the impact of migraine/headaches on daily activities, quality of life, family, and work was collected using validated patient-reported outcome instruments, including HIT-6 (over the past 4 weeks, where relevant), MIBS-4 (over the past 4 weeks), and MIDAS (over the past 3 months), described above. Total MIDAS scores are categorized into four grades: Grade I: little or no disability; Grade II: mild disability; Grade III: moderate disability; Grade IV: severe disability [8, 36]. MIDAS includes a question on headache pain severity on a scale of 0–10, which was separately analyzed in this study. Additional instruments included the following: Migraine-Specific Quality-of-Life Questionnaire (MSQ) version 2.1, a 14-item questionnaire with three domains (Role Function-Restrictive, Role Function-Preventive, Emotional Function) that addresses physical and emotional limitations associated with migraine over the past 4 weeks [37]; Impact of Migraine on Partners and Adolescent Children Scale (IMPAC), a 12-item questionnaire measuring the impact of migraine on the family (previous 30 days) [38], completed only by participants who were living with a partner/spouse and/or children (total IMPAC scores are categorized into four grades: Grade I: no or mild impact; Grade II: moderate impact; Grade III: severe impact; Grade IV: very severe impact); and Work Productivity and Activity Impairment Questionnaire-Migraine (WPAI-M), a six-item questionnaire with four domains (Activity Impairment, Absenteeism, Presenteeism, Work Productivity Loss) over the past 7 days [39]; all participants completed the Activity Impairment domain, but only employed participants completed the other three domains. Validated Japanese versions of the HIT-6, MIDAS, and MSQ were used, whereas the ACS-12, MIBS-4, WPAI-M, and IMPAC were translated from English using standard (forward and backward) translation. Participants were also asked how often (“never”, “rarely”, “sometimes”, “often”, or “always”) over the past 3 months they worried about or were careful to avoid migraines/headaches when they did not have a headache.

Participants were also asked to estimate costs (Japanese yen [JPY] per month) over the past 3 months associated with migraine/headaches in each of the following categories: medical consultations and prescribed medications; over-the-counter (OTC) headache drugs; transportation to and from medical facilities; supplements, food, etc.; massage, physical therapy, gym, etc.; items for headache (pillows, fragrances, sunglasses, etc.); eating out, take-out food for participant and their family during headaches; taxi fares during headaches; transportation of children during headaches; babysitting during headaches; gifts of gratitude to people for their help; and other costs. The results were analyzed for each category separately, as well as for the total sum of estimated expenses.

Participant medical experiences for headaches at any time were assessed, including physician consultation (specialist or non-specialist), diagnosis of migraine, and whether they were currently attending a medical institution for migraine/headaches at the time of the survey. Participant experiences with drug treatment at any time were also assessed; this included experience using OTC drugs and prescribed acute treatments, specifically, analgesics (non-steroidal anti-inflammatory drugs and acetaminophen), triptans, lasmiditan, oral preventive drugs, and anti-CGRP mAbs. Participants who had used OTC and/or prescribed acute treatment for headache within the past year rated their satisfaction with acute treatment using the Migraine Treatment Optimization Questionnaire-6 (mTOQ-6), a six-item questionnaire that measures the effectiveness of current acute migraine treatment during the past 3 months [40]; a translated version of the mTOQ was used. Participants who had experience with preventive medication indicated the benefits of using these drugs from a predefined list.

### Statistical analysis

Survey results were analyzed for the overall migraine group and for subgroups based on HIT-6 and MIBS-4 scores (Table 1). The HIT-6 was used as an indicator of ictal burden instead of MIDAS because HIT-6 places less emphasis on absenteeism than MIDAS. Evidence suggests that absenteeism is lower in Japanese patients with migraine compared with non-Japanese patients, which is likely the result of differing cultural norms regarding work in Japan [22, 29]. Therefore, we considered the HIT-6 to be a more appropriate measure of ictal burden than MIDAS in our cohort. HIT-6 scores ≥ 60 (indicating severe impact during headaches [41]) were defined as “higher HIT-6”, and HIT-6 scores < 60 (indicating little or no impact, some impact, or substantial impact [41]) were defined as “lower HIT-6”. MIBS-4 scores ≥ 3 (indicating moderate or severe impact between headache attacks [12]) were defined as “higher MIBS-4”, and MIBS-4 scores < 3 (indicating no or mild impact [12]) were defined as “lower MIBS-4”. Four subgroups were defined based on HIT-6 and MIBS-4 scores: the “Severe” subgroup had higher HIT-6 and higher MIBS-4, the “High Interictal Burden” subgroup had lower HIT-6 and higher MIBS-4, the “High Ictal Burden” subgroup had higher HIT-6 and lower MIBS-4, and the “Milder” subgroup had lower HIT-6 and lower MIBS-4. The Severe and High Interictal Burden subgroups were considered “MIBS-High” subgroups, and the High Ictal Burden and Milder subgroups were considered “MIBS-Low” subgroups. Similarly, the Severe and High Ictal Burden subgroups were considered “HIT-High” subgroups, and the High Interictal Burden and Milder subgroups were considered “HIT-Low” subgroups.

**Table 1:** Subgroup definitions based on HIT-6 and MIBS-4 scores

**Subgroup family**	**Subgroup**	**HIT-6 score**	**MIBS-4 score**
*Primary analysis*			
Main subgroups	Severe	HIT-High: ≥ 60	MIBS-High: ≥ 3
	High Interictal Burden	HIT-Low: < 60	MIBS-High: ≥ 3
	High Ictal Burden	HIT-High: ≥ 60	MIBS-Low: < 3
	Milder	HIT-Low: < 60	MIBS-Low: < 3
*Additional analyses*			
HIT-6 subgroups	Severe	60–78	Any
	Substantial	56–59	Any
	Moderate	50–55	Any
	Little-to-none	36–49	Any
MIBS-4 subgroups	Severe	Any	≥ 5
	Moderate	Any	3–4
	Mild	Any	1–2
	None	Any	0

*Abbreviations*: *HIT-6* Headache Impact Test-6, *MIBS-4* Migraine Interictal Burden Scale-4

Mean and standard deviation (SD) were calculated for continuous variables, and number (%) calculated for categorical variables. Differences in baseline characteristics across subgroups were compared using analysis of variance for continuous variables and chi-square tests for categorical variables. Pairwise comparisons between subgroups were made using *t* tests for continuous variables and chi-square tests for categorical variables, except for pain duration and frequency of concern, which were compared between pairs of subgroups using a Mann–Whitney U test. No multiplicity adjustments were made in the statistical analyses. *p*-values < 0.05 were considered statistically significant. Python version 3.9.7 (Python Software Foundation, Wilmington, DE, USA) and BellCurve for Excel version 4.04 or later (Social Survey Research Information Co., Ltd., Tokyo, Japan) were used for statistical analyses.

In addition to the four main subgroups, other subgroups based only on HIT-6 or MIBS-4 scores were assessed (Table 1). The HIT-6 subgroups based on level of impact were “Little-to-none” (scores 36–49), “Moderate” (scores 50–55), “Substantial” (scores 56–59), and “Severe” (scores 60–78) [41]. The MIBS-4 subgroups based on level of impact were “None” (score of 0), “Mild” (scores 1–2), “Moderate” (scores 3–4), and “Severe” (scores ≥ 5) [12]. Results for the HIT-6 only and MIBS-4 only subgroups are descriptive only, and no statistical comparisons between subgroups were made.

## Results

### Participant characteristics

Of 1,423,573 potential respondents who were invited to participate, 19,590 individuals met the eligibility criteria for migraine and completed the survey (Figure S1). Within the overall migraine population, 6854 (35.0%) were in the Severe subgroup, 6115 (31.2%) were in the Milder subgroup, 4253 (21.7%) were in the High Ictal Burden subgroup, and 2368 (12.1%) were in the High Interictal Burden subgroup (Figure S1, Table 2).

**Table 2:** Demographics and clinical characteristics

**Variable**	**Overall**^**a**^ **(*N* = 19,590)**	**MIBS-High**		**MIBS-Low**		***p*-value**^**b**^
		**Severe** **(*N* = 6854)**	**High Interictal Burden** **(*N* = 2368)**	**High Ictal Burden** **(*N* = 4253)**	**Milder** **(*N* = 6115)**	
MIBS-4 score, mean (SD)	3.2 (3.6)	6.9 (2.6)	5.5 (2.1)	0.4 (0.7)	0.2 (0.6)	< 0.001
HIT-6 score, mean (SD)	59.7 (7.8)	65.5 (4.9)	54.9 (3.9)	64.2 (4.2)	52.0 (5.3)	< 0.001
Age, years, mean (SD)	40.5 (13.1)	38.7 (12.3)	40.6 (13.7)	39.7 (12.2)	43.1 (14.0)	< 0.001
Female sex, *n* (%)	13,486 (68.8)	4666 (68.1)	1375 (58.1)	3299 (77.6)	4146 (67.8)	< 0.001
Employment, *n* (%)						< 0.001
Full-time employee	7769 (39.7)	2854 (41.6)	1119 (47.3)	1526 (35.9)	2270 (37.1)	
Part-time, temporary, or contract employee	4955 (25.3)	1672 (24.4)	522 (22.0)	1150 (27.0)	1611 (26.3)	
Self-employed	877 (4.5)	310 (4.5)	93 (3.9)	196 (4.6)	278 (4.5)	
Stay-at-home spouse	2304 (11.8)	663 (9.7)	230 (9.7)	626 (14.7)	785 (12.8)	
Student with part-time job	699 (3.6)	267 (3.9)	95 (4.0)	140 (3.3)	197 (3.2)	
Student with no part-time job	349 (1.8)	135 (2.0)	42 (1.8)	73 (1.7)	99 (1.6)	
On long- or short-term leave	140 (0.7)	57 (0.8)	16 (0.7)	31 (0.7)	36 (0.6)	
Unemployed, seeking employment	702 (3.6)	263 (3.8)	65 (2.7)	158 (3.7)	216 (3.5)	
Unemployed, not seeking employment	1288 (6.6)	431 (6.3)	120 (5.1)	254 (6.0)	483 (7.9)	
Prefer not to answer	507 (2.6)	202 (2.9)	66 (2.8)	99 (2.3)	140 (2.3)	
Marriage status, *n* (%)						< 0.001
Single, never married	8273 (42.2)	3128 (45.6)	1021 (43.1)	1675 (39.4)	2449 (40.0)	
Married	8881 (45.3)	2850 (41.6)	1095 (46.2)	2044 (48.1)	2892 (47.3)	
Living with a partner	816 (4.2)	334 (4.9)	90 (3.8)	183 (4.3)	209 (3.4)	
Divorced	1263 (6.4)	418 (6.1)	126 (5.3)	272 (6.4)	447 (7.3)	
Widowed	215 (1.1)	64 (0.9)	20 (0.8)	52 (1.2)	79 (1.3)	
Prefer not to answer	142 (0.7)	60 (0.9)	16 (0.7)	27 (0.6)	39 (0.6)	
Cohabitation status^c^* n* (%)						
Live alone	4264 (21.8)	1598 (23.3)	537 (22.7)	853 (20.1)	1276 (20.9)	< 0.001
Spouse/partner	9083 (46.4)	2950 (43.0)	1105 (46.7)	2114 (49.7)	2914 (47.7)	< 0.001
Children/stepchildren/grandchildren	6333 (32.3)	2153 (31.4)	742 (31.3)	1544 (36.3)	1894 (31.0)	< 0.001
Parents/parents-in-law	5666 (28.9)	2095 (30.6)	668 (28.2)	1146 (26.9)	1757 (28.7)	< 0.001
Other people	1975 (10.1)	698 (10.2)	243 (10.3)	401 (9.4)	633 (10.4)	0.45
Disease history during past 12 months^c^* n* (%)						
Dizziness	5769 (29.4)	2384 (34.8)	603 (25.5)	1279 (30.1)	1503 (24.6)	< 0.001
Sinusitis	2195 (11.2)	909 (13.3)	287 (12.1)	470 (11.1)	529 (8.7)	< 0.001
Insomnia	4220 (21.5)	2001 (29.2)	455 (19.2)	853 (20.1)	911 (14.9)	< 0.001
Depression/depressive state	3118 (15.9)	1631 (23.8)	292 (12.3)	599 (14.1)	596 (9.7)	< 0.001
Anxiety neurosis/anxiety disorder/panic disorder	2317 (11.8)	1238 (18.1)	234 (9.9)	430 (10.1)	415 (6.8)	< 0.001
Constipation	5133 (26.2)	2017 (29.4)	541 (22.8)	1180 (27.7)	1395 (22.8)	< 0.001
Chronic lower back pain	3320 (16.9)	1323 (19.3)	346 (14.6)	750 (17.6)	901 (14.7)	< 0.001
Stiff shoulders	11,395 (58.2)	4075 (59.5)	1265 (53.4)	2606 (61.3)	3449 (56.4)	< 0.001

*Abbreviations*: *HIT-6* Headache Impact Test-6, *MIBS-4* Migraine Interictal Burden Scale-4, *SD* standard deviation

^a^Data (except disease history) for the overall population also reported in Danno et al. [32]

^b^*p*-value across subgroups using analysis of variance for continuous variables (MIBS-4, HIT-6, age) and chi-square test for categorical variables

^c^More than one answer possible

Significant differences in almost all baseline characteristics were observed across the four subgroups (Table 2). The High Ictal Burden subgroup tended to have a greater proportion of women and a greater proportion living with a spouse/partner and/or children compared with the other subgroups. Conversely, the High Interictal Burden subgroup had a lower proportion of women and a greater proportion of full-time employees than the other subgroups. Comorbidities were usually most common in the Severe subgroup and least common in the Milder subgroup. In general, within each HIT-6 level, a greater proportion of respondents in the subgroups with higher MIBS-4 scores had comorbidities compared with the subgroups with lower MIBS-4 scores.

In the subgroups based on HIT-6 score only, the proportion of women and the proportion of respondents with comorbidities tended to increase with increasing HIT-6 score (Table S1). In the subgroups based on MIBS-4 score only, the proportion of full-time employees and the proportion of respondents with comorbidities tended to increase with increasing MIBS-4 score (Table S2).

### Headache symptoms and allodynia

**Fig. 1:**
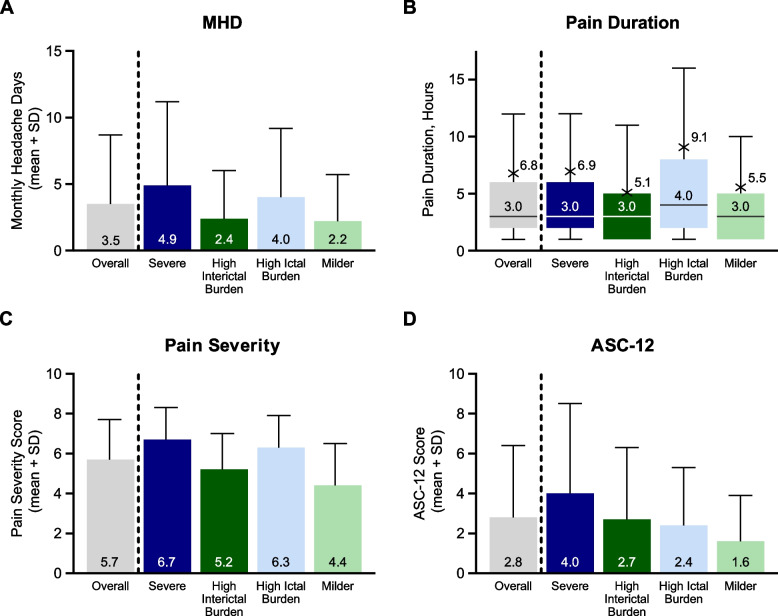
Headache symptoms and allodynia in the overall cohort and in the Severe, High Interictal Burden, High Ictal Burden, and Milder subgroups. HIT-High subgroups are shown in blue, and HIT-Low subgroups are shown in green. MIBS-High subgroups are shown in dark colors, and MIBS-Low subgroups are shown in light colors. **A** MHD. **B** Pain duration. As the distribution of pain duration was skewed, these data are presented as boxplots (the horizontal line within each box represents the median, the cross represents the mean, the bottom and top of each box represent the 25th and 75th percentile, respectively, and the error bars represent the range, excluding outliers [outside 1.5 times the interquartile range above the 75th percentile and below the 25th percentile]). **C** Pain severity. **D** ASC-12 scores. For all variables, *p* < 0.01 (*t* test for MHD, pain severity, and ASC-12 scores; Mann–Whitney U test for pain duration) for four pairwise comparisons between HIT-High subgroups vs. HIT-Low subgroups (Severe vs. High Interictal Burden or Milder subgroups, and High Ictal Burden vs. High Interictal Burden or Milder subgroups). Abbreviations: ASC-12, Allodynia Symptoms Checklist-12; HIT, Headache Impact Test; MHD, monthly headache days; MIBS, Migraine Interictal Burden Scale; SD, standard deviation

Symptoms that are directly related to migraine headaches, such as the mean number of monthly headache days and mean headache pain severity score, were higher in HIT-High subgroups than in HIT-Low subgroups (Fig. 1A, C). In addition, the median headache pain duration was higher in the High Ictal Burden group than in the other subgroups (Fig. 1B). In contrast, the frequency of allodynia symptoms (ASC-12 score) was higher in the High Interictal Burden subgroup than in the High Ictal Burden subgroup (Fig. 1D).

In the subgroups based on HIT-6 score only, monthly headache days, pain duration, pain severity, and ASC-12 score tended to increase with increasing HIT-6 score (Figure S2). In the subgroups based on MIBS-4 score only, monthly headache days, pain severity, and ASC-12 score, but not pain duration, tended to increase with increasing MIBS-4 score (Figure S3). In addition, mean ASC-12 score was numerically higher in the highest MIBS-4 subgroup than in the highest HIT-6 subgroup, supporting the association of allodynia symptoms with the interictal period.

### Health-related quality of life, daily activities, work, and family

**Fig. 2:**
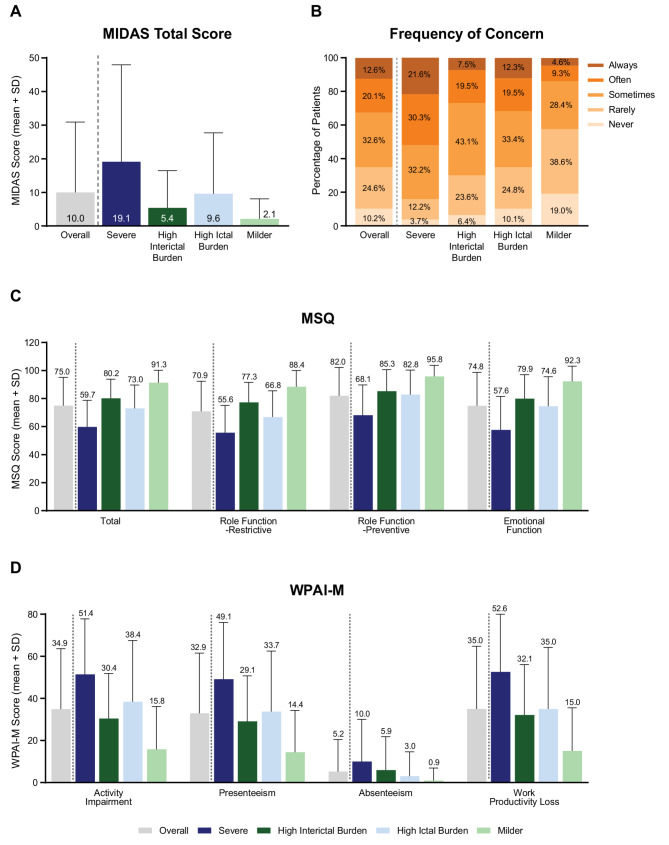
**A** MIDAS total score. **B** Frequency of concern about headaches during the interictal period. **C** MSQ score. **D** WPAI-M score among all participants (Activity Impairment) or participants who are employed (overall *n* = 11,891 for Presenteeism and Work Productivity Loss; overall *n* = 11,981 for Absenteeism). HIT-High subgroups are shown in blue, and HIT-Low subgroups are shown in green. MIBS-High subgroups are shown in dark colors, and MIBS-Low subgroups are shown in light colors. For all variables, *p* < 0.01 (*t* test for MIDAS, MSQ, and WPAI-M; Mann–Whitney U test for frequency of concern) for all six pairwise comparisons between subgroups (Severe vs. High Interictal Burden, High Ictal Burden, or Milder subgroups, High Ictal Burden vs. High Interictal Burden or Milder subgroups, and High Interictal Burden vs. Milder subgroups), except for frequency of concern between High Interictal Burden vs. High Ictal Burden. Abbreviations: HIT, Headache Impact Test; MIBS, Migraine Interictal Burden Scale; MIDAS, Migraine Disability Assessment; MSQ, Migraine-Specific Quality-of-Life Questionnaire; SD, standard deviation; WPAI-M, Work Productivity and Activity Impairment-Migraine

**Fig. 3:**
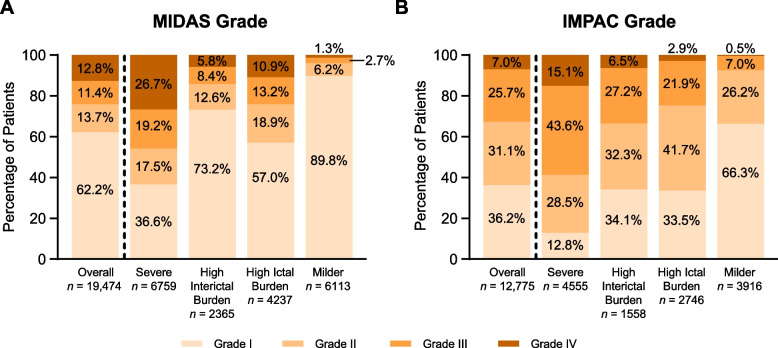
Distribution of **A** MIDAS grade (overall *n* = 19,474) and **B** IMPAC grade (among participants with partners/spouses and/or children under 8 years of age; overall *n* = 12,775) in the overall cohort and in the Severe, High Interictal Burden, High Ictal Burden, and Milder subgroups. Abbreviations: IMPAC, Impact of Migraine on Partners and Adolescent Children Scale; MIDAS, Migraine Disability Assessment

The total MIDAS score was higher in HIT-High subgroups than in HIT-Low subgroups (Fig. 2A) and tended to increase with increasing HIT-6 score (Figure S4). In addition, within both HIT-High and HIT-Low subgroups, total MIDAS score was higher in MIBS-High subgroups (Fig. 2A). Similar trends were observed for MIDAS grade (Fig. 3A, Figure S5, Figure S6). IMPAC grade, which reflects the impact of migraine on family members, was higher in the High Interictal Burden subgroup than in the High Ictal Burden subgroup (Fig. 3B; proportion with Grade III or IV, 33.6% vs. 24.8%), with a significant difference across subgroups in the proportion of Grade I or II versus Grade III or IV (*p* < 0.001, chi-square test). In subgroups based on HIT-6 or MIBS-4 only, IMPAC grade increased with both increasing HIT-6 (Figure S5) and increasing MIBS-4 scores (Figure S6).

MIBS-High subgroups were concerned about headaches during the interictal period more frequently than MIBS-Low subgroups (Fig. 2B), with the frequency of concern increasing with increasing MIBS-4 score (Figure S7). Notably, despite both being MIBS-Low subgroups, the High Ictal Burden subgroup had more frequent interictal concerns than the Milder subgroup. MSQ scores were generally lower (i.e. lower quality of life) in HIT-High subgroups than in HIT-Low subgroups (Fig. 2C) and tended to decrease with increasing HIT-6 score (Figure S7). In addition, within both HIT-High and HIT-Low subgroups, those with higher MIBS-4 scores had lower MSQ scores (Fig. 2C). The degree of work and activity impairment, as indicated by WPAI-M scores, showed patterns similar to the MIDAS and MSQ scores, except that more absenteeism was observed in the High Interictal Burden subgroup than in the High Ictal Burden subgroup (Fig. 2D).

### Economic burden

In the overall migraine group, the mean total cost of migraine was 3861.7 JPY per month (Table 3). The total economic burden associated with migraine increased with both increasing HIT-6 scores (Table S3) and increasing MIBS-4 scores (Table S4). Within both HIT-High and HIT-Low subgroups, costs were greater in MIBS-High subgroups than in MIBS-Low subgroups (Table 3). In particular, the total cost of migraine was significantly higher in the High Interictal Burden subgroup than in the High Ictal Burden subgroup (mean costs of 3578.3 and 2816.6 JPY/month, respectively). Most of the higher costs in the High Interictal Burden subgroup were related to medical consultations (including costs associated with transportation to and from medical institutions), prescribed medications, OTC drugs, and supplements.

**Table 3:** Economic burden of migraine

**Expense, JPY/Month, Mean (95% CI)**	**Overall** **(*N* = 19,590)**	**MIBS-High**		**MIBS-Low**	
		**Severe** **(*N* = 6854)**	**High Interictal Burden** **(*N* = 2368)**	**High Ictal Burden** **(*N* = 4253)**	**Milder** **(*N* = 6115)**
Consultations and medications prescribed by the doctor	936.5 (874.0–999.0)	1681.8 (1516.3–1847.4)	843.8 (746.3–941.4)	671.9 (599.9–743.9)	320.9 (287.5–354.3)
Transportation to and from medical institutions	219.6 (190.6–248.5)	432.1 (355.5–508.7)	225.3 (147.9–302.6)	105.4 (89.8–121.1)	58.5 (45.4–71.6)
OTC headache drugs	794.3 (727.9–860.7)	1081.7 (1002.3–1161.2)	949.4 (526.8–1372.0)	708.8 (666.4–751.3)	471.4 (373.5–569.3)
Supplements, food, etc	465.0 (385.9–544.0)	868.2 (658.1–1078.3)	506.5 (357.1–655.8)	254.7 (184.9–324.6)	143.1 (89.7–196.5)
Massage, physical therapy, going to a gym, etc	477.4 (434.1–520.7)	721.9 (637.6–806.2)	432.8 (339.3–526.4)	416.0 (346.4–485.5)	263.5 (182.1–344.8)
Items for headache (pillows, fragrances, sunglasses, etc.)	135.7 (114.0–157.4)	264.6 (206.2–323.0)	98.4 (73.2–123.6)	89.8 (65.6–114.0)	37.6 (25.9–49.4)
Eating out, take-out (prepared foods, boxed lunches), and catering (delivery) for myself and my family during headaches	465.1 (419.2–510.9)	864.1 (749.4–978.8)	251.5 (195.4–307.6)	445.0 (357.6–532.3)	114.5 (87.8–141.3)
Taxi fares during headaches	113.2 (76.1–150.3)	263.6 (159.1–368.1)	64.1 (39.9–88.2)	42.8 (20.7–65.0)	12.5 (5.2–19.9)
Transportation of children during my headaches	35.3 (21.3–49.3)	83.2 (44.1–122.2)	16.8 (7.9–25.6)	14.6 (2.6–26.5)	3.2 (–0.5, 6.9)
Babysitting at home or elsewhere during a headache	54.5 (29.7–79.2)	130.1 (60.7–199.5)	29.0 (11.5–46.6)	20.2 (0.7–39.7)	3.3 (–0.4, 7.0)
Gifts of my gratitude to people for their help	83.2 (61.2–105.1)	190.7 (129.9–251.5)	61.5 (30.0–93.1)	28.3 (15.0–41.5)	9.1 (1.4–16.8)
Other	82.2 (29.7–134.6)	121.2 (30.4–211.9)	99.2 (9.0–189.3)	19.1 (2.0–36.1)	75.7 (–52.9, 204.3)
**Total**^**a**^	**3861.7 (3634.4–4089.0)**	**6703.2 (6136.9–7269.6)**	**3578.3 (3032.6–4123.9)**	**2816.6 (2583.7–3049.4)**	**1513.4 (1296.6–1730.2)**

Totals are shown in bold

At the time of the study, 1000 JPY was approximately equivalent to US$7.00 and €6.40

*Abbreviations: CI* confidence interval, *JPY* Japanese yen, *MIBS* Migraine Interictal Burden Scale, *OTC* over-the-counter

^a^*p* < 0.01 (*t* test) for pairwise comparisons of total cost between Severe vs. High Ictal Burden, Severe vs. Milder, High Interictal Burden vs. High Ictal Burden, and High Interictal Burden vs. Milder subgroups

### Medical and drug treatment

**Fig. 4:**
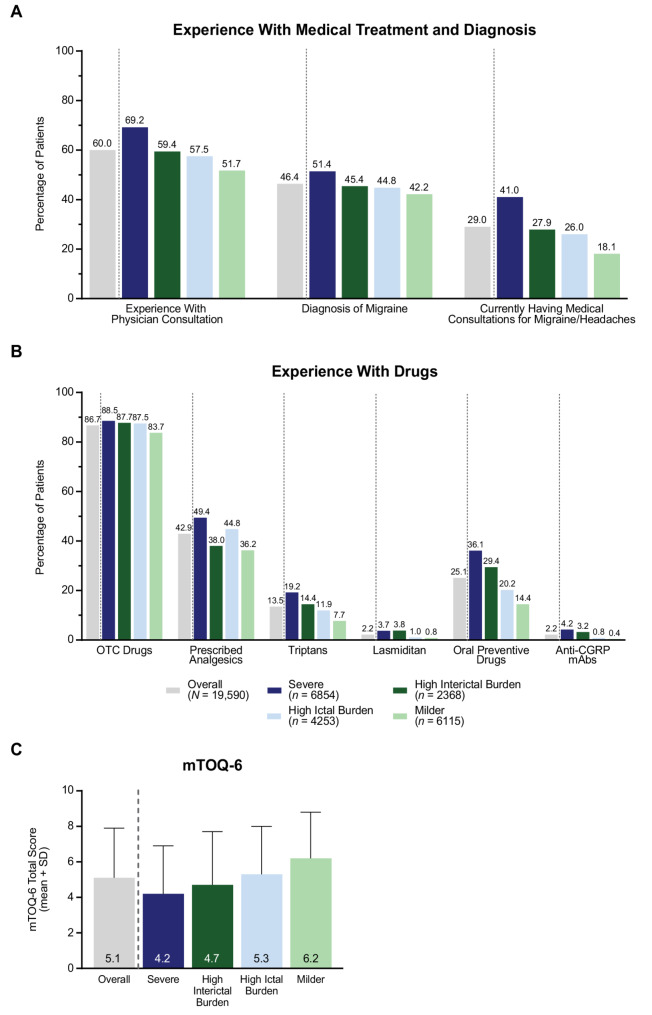
**A** Experience with medical treatment and diagnosis. **B** Experience with drugs. **C** mTOQ-6 score (in participants who had used OTC and/or prescription acute treatment for headache within the past year; overall *n* = 17,094). HIT-High subgroups are shown in blue, and HIT-Low subgroups are shown in green. MIBS-High subgroups are shown in dark colors, and MIBS-Low subgroups are shown in light colors. For all variables, *p* < 0.01 (chi-square test, except *t* test for mTOQ-6) for all four pairwise comparisons between HIT-High subgroups vs. HIT-Low subgroups (Severe vs. High Interictal Burden or Milder subgroups, and High Ictal Burden vs. High Interictal Burden or Milder subgroups), except for experience with physician consultation between High Interictal Burden vs. High Ictal Burden, migraine diagnosis between High Interictal Burden vs. High Ictal Burden, currently having medical consultations between High Interictal Burden vs. High Ictal Burden, OTC use between Severe vs. High Ictal Burden and between High Interictal Burden and High Ictal Burden, and prescribed analgesics between High Interictal Burden and Milder. Abbreviations: CGRP, calcitonin gene-related peptide; HIT, Headache Impact Test; mAbs, monoclonal antibodies; MIBS, Migraine Interictal Burden Scale; mTOQ-6, Migraine Treatment Optimization Questionnaire-6; OTC, over-the-counter; SD, standard deviation

Most (60.0%) participants in the overall migraine group had experience with physician consultation for headaches (Fig. 4A). Approximately half (46.4%) of participants had received a diagnosis of migraine, and 29.0% were currently having medical consultations for their headaches. The proportion of participants with these experiences was notably higher in the Severe subgroup compared with the other subgroups; this pattern was also observed in subgroups based on HIT-6 or MIBS-4 score only (Figure S8, Figure S9). While the proportion of participants with these experiences was generally higher in MIBS-High subgroups than in MIBS-Low subgroups, the proportions were similar between those in High Ictal Burden and High Interictal Burden subgroups.

Overall, most participants in all subgroups had used OTC drugs (Fig. 4B). Use of acute treatment prescription analgesics was more common in HIT-High subgroups than in HIT-Low subgroups (Fig. 4B) and tended to increase with increasing HIT-6 score (Figure S8). However, triptans and lasmiditan were more frequently used in the High Interictal Burden subgroup than in the High Ictal Burden subgroup; these drugs were also most commonly used in the highest (Severe) MIBS-4 subgroup (Figure S9). Preventive medications, including oral preventive drugs and anti-CGRP mAbs, were also more commonly used in the High Interictal Burden subgroup than in the High Ictal Burden subgroup.

Among participants who used acute treatment drugs (OTC and/or prescription), satisfaction with acute treatment was lower in MIBS-High subgroups than in MIBS-Low subgroups (Fig. 4C) and tended to decrease with increasing MIBS-4 score (Figure S9). Mean (SD) mTOQ-6 scores were 4.2 (2.7) and 4.7 (3.0) in the Severe (*n* = 6206) and High Interictal Burden (*n* = 2017) subgroups, respectively, compared with 5.3 (2.7) and 6.2 (2.6) in the High Ictal Burden (*n* = 3832) and Milder (*n* = 5039) subgroups.

Among participants who had used preventive medications (*n* = 2628), the most common benefits (> 30% of participants in the overall migraine group) were “less frequent headache drug doses”, “more headache-free days”, and “milder headaches” (Figure S10). Interestingly, the benefits that were more common in MIBS-High subgroups than in MIBS-Low subgroups were “able to do things that I could not”, “increased work efficiency”, “no more missed work”, “fewer visits to the doctor”, and “enjoy time with family”.

## Discussion

This analysis of the OVERCOME (Japan) 2nd study indicates that interictal burden, as measured by MIBS-4 scores, has a substantial impact on the daily lives of Japanese people with migraine that is independent of the impact of headaches (HIT-6 scores). In this study, almost half (47.1%) of respondents with migraine had MIBS-4 scores ≥ 3, indicating moderate to severe interictal burden. Comparable proportions of moderate to severe interictal burden were reported in the OVERCOME (US) (53.8%) [16] and OVERCOME (EU) (12.6% moderate, 41.2%–57.8% severe) [14] studies, as well as in a separate survey conducted in the US and Germany (67% severe) [15]. Moreover, 12.1% of respondents in the current study had high interictal burden but not high ictal burden. Despite having milder headache symptoms, the High Interictal Burden subgroup reported a greater impact of migraine on family, more allodynia, higher migraine-associated costs, and similar levels of some comorbidities and migraine impact on work and quality of life compared with the High Ictal Burden subgroup. Overall, these results confirm that the impact of migraine on daily life extends beyond headache attacks, and interictal burden should be considered as part of the management and treatment of migraine.

In general, participants with higher MIBS-4 scores experienced a greater negative impact of migraine on daily life than participants with lower MIBS-4 scores, even among those with lower HIT-6 scores. Both ictal and interictal burden contributed to overall disability and quality of life, as evidenced by the higher MIDAS score and lower MSQ scores, respectively, among the subgroups with higher MIBS-4 scores within each HIT-6 category. However, some aspects of daily life were more affected by interictal burden than by ictal burden. For example, the impact on family (IMPAC grade) was greater in the High Interictal Burden subgroup than in the High Ictal Burden subgroup. This result may be related to anxiety or fear about how the next migraine headache could affect partners and children [4, 7]. Work was also negatively affected by interictal burden; within each HIT-6 category, a higher MIBS-4 score was associated with greater activity impairment, productivity loss, absenteeism, and presenteeism. Considering that previous Japanese studies have established the economic cost associated with absenteeism and presenteeism due to migraine [42, 43], our results indicate that interictal burden may also contribute substantially to this economic loss due to migraine. Interestingly, the High Interictal Burden subgroup had a greater proportion of men and full-time employees than the other subgroups; thus, working men may have more interictal burden than other demographic groups through negative work- or employment-related experiences due to migraine even between migraine attacks.

Consistent with their high interictal burden, participants in MIBS-High subgroups were more frequently concerned about their headaches during the interictal period than those in MIBS-Low subgroups. Unexpectedly, despite both subgroups having lower MIBS-4 scores, participants in the High Ictal Burden subgroup reported more frequent concerns between headaches than the Milder subgroup. This result suggests that there may be aspects of interictal burden that are not adequately captured by the MIBS-4 alone. One possible reason for this is that patients with frequent and/or prolonged headaches may have limited time between headaches, which may hinder the ability of the MIBS-4 to assess interictal burden accurately in these patients. Although the MIBS-4 is a simple and convenient tool to evaluate interictal burden, physicians should be mindful of its potential limitations and use a range of approaches to determine the relative contributions of ictal and interictal burden in individual patients.

Within each HIT-6 category, participants with higher MIBS-4 scores reported higher personal costs associated with migraine, and more experience with medical consultation and drug treatments, than those with lower MIBS-4 scores. Most of the higher costs were related to treatments and medical visits. This result may suggest that people with higher interictal burden experience significant migraine-related burden across many aspects of their lives and are actively seeking medical treatment. This observation may also be consistent with the results of the OVERCOME (US) study, which found that interictal burden was one of the strongest factors associated with seeking medical care [13].

Despite their active attitude in seeking medical care and extent of experience with acute medications for migraine, satisfaction with these treatments (mTOQ-6 score) was lower in the High Interictal Burden subgroup compared with the High Ictal Burden subgroup, indicating that outcomes from medical management are not always satisfactory. Moreover, a lack of efficacy of acute treatments may lead to greater worry about the next headache, thereby contributing to interictal burden. These results suggest that people with higher interictal burden may be more likely to have migraine that requires not only acute medications but also preventive medications. In the current analysis, the Severe and High Interictal Burden subgroups had more experience with preventive medications than the High Ictal Burden and Milder subgroups, suggesting that those with higher interictal burden were more likely to seek and use preventive drugs. In 2023, when the OVERCOME (Japan) 2nd study was conducted, the only preventive drugs available in Japan were conventional oral preventive drugs and anti-CGRP mAbs; the oral gepants are expected to become available in the future. The availability of several preventive drug options will help address the personal needs of individual patients and will likely increase the number of people who try at least one preventive medication. Preventive drugs have been shown to reduce interictal burden [17] and improve cognitive function during the interictal period [44, 45]. Importantly, when asked about the benefits of preventive drugs, participants with higher interictal burden were more likely to cite benefits related to minimizing disruptions to work and family life compared with participants with lower interictal burden. Because Japanese people with migraine are often motivated to reduce the impact of headaches on their lives [46, 47], it is important for physicians to understand their individual needs, including the level of interictal burden, which will assist in selecting the most appropriate treatment.

Although headache-related symptoms, such as number of monthly headache days, pain severity, and headache duration, aligned with HIT-6 scores as expected, allodynia symptoms (ASC-12 scores) tended to be more frequent in participants with higher interictal burden. There is evidence that allodynia, as well as hyperalgesia, is present in both the ictal and interictal periods [48, 49] and may be a predictor of progression to chronic migraine [50]. In the OVERCOME (Japan) 2nd study, ASC-12 scores increased with increasing number of monthly headache days [32]. However, how allodynia contributes to interictal burden needs further investigation [7]. Functional magnetic resonance imaging studies have demonstrated that people with migraine have altered somatosensory processing during the interictal period that may be related to the experience of allodynia [51, 52]. This hypersensitivity may contribute to interictal burden by making patients more aware of their migraine between headaches. In addition, patients with allodynia may be less responsive to some preventive treatments [53], further increasing their interictal burden.

The OVERCOME (Japan) 2nd study was a nationwide survey of almost 20,000 Japanese people with migraine. The study sample was demographically matched to the general Japanese population, and a broad range of outcome measures was used to evaluate the impact of migraine in subgroups based on HIT-6 (ictal burden) and MIBS-4 (interictal burden) scores. Despite these strengths, several limitations must be acknowledged. As this was an online survey, there was potential for selection bias and recall bias, and the cross-sectional study design precludes any longitudinal analysis. Although we categorized participants with HIT-6 scores < 60 as having “lower HIT-6”, scores in the range of 50–60, as seen in the High Interictal Burden and Milder subgroups, indicate moderate to substantial impact of headaches [41]. However, to ensure sufficient numbers of participants in each subgroup, we elected to use the higher HIT-6 cutoff. In addition, interictal and ictal burden levels were not analyzed based on frequency of headaches (e.g. chronic vs. episodic migraine) or type of drug treatment. One of the MIBS-4 questions asks about the effect of headaches on work or school during the interictal period, which would not be applicable to participants who are not employees or students; this may have contributed to some of the demographic differences between the High Interictal Burden and High Ictal Burden subgroups. Finally, the estimation of costs associated with migraine was subject to recall bias, which could have been compounded when estimating total costs by summing the costs for individual categories.

## Conclusions

This analysis of the OVERCOME (Japan) 2nd study revealed that high interictal burden negatively affects multiple aspects of daily life in Japanese people with migraine independently of the impact of headaches. In particular, our results reveal a greater impact of interictal burden on family, migraine-associated costs, allodynia, and satisfaction with acute treatments than is typically recognized. Although interictal burden may not be routinely assessed in clinical practice, its measurement using instruments such as the MIBS-4 will help physicians better understand the burdens associated with migraine and improve the treatment of people with migraine.

## Supplementary Information

Supplementary Material 1.

## Data Availability

The datasets generated during and/or analyzed during the current analysis are available from the corresponding author on reasonable request.

## Abbreviations

ASC-12: Allodynia Symptom Checklist-12
HIT-6: Headache Impact Test-6
ICHD-3: International Classification of Headache Disorders, 3rd edition
IMPAC: Impact of Migraine on Partners and Adolescent Children Scale
JPY: Japanese yen
mAbs: Monoclonal antibodies
MIBS-4: Migraine Interictal Burden Scale-4
MIDAS: Migraine Disability Assessment
MSQ: Migraine-Specific Quality-of-Life Questionnaire
mTOQ-6: Migraine Treatment Optimization Questionnaire-6
OTC: Over-the-counter
OVERCOME: ObserVational survey of the Epidemiology, tReatment, and Care Of MigrainE
SD: Standard deviation
WPAI-M: Work Productivity and Activity Impairment Questionnaire-Migraine
