# Supplementary material for: The dual impact of ictal and interictal burden in migraine: an analysis from the ObserVational survey of the Epidemiology, tReatment, and Care Of MigrainE (OVERCOME) Japan second study
Source: J Headache Pain. 2025 Jun 16;26(1):140. doi: 10.1186/s10194-025-02079-z (PMC12168418; doi:10.1186/s10194-025-02079-z)

Supplementary Information

TITLE

The dual impact of ictal and interictal burden in migraine: an analysis from the ObserVational survey of the Epidemiology, tReatment, and Care Of MigrainE (OVERCOME) Japan second study

Tsubasa Takizawa[1], Daisuke Danno[2], Ryotaro Ishii[3], Shiho Suzuki[4], Moemi Miura[5], Yoshinori Tanizawa[6*], Satoshi Osaga[6], Michio Okada[6], Chie Hashimoto[6] and Mika Komori[6]

AUTHORS' AFFILIATIONS

[1]Department of Neurology, Keio University School of Medicine, Tokyo, Japan

[2]Headache Center and Department of Neurology, Tominaga Hospital, Osaka, Japan

[3]Department of Neurology, Kyoto Prefectural University of Medicine, Kyoto, Japan

[4]Department of Neurology, Dokkyo Medical University, Tochigi, Japan

[5]Social Survey Research Information Co., Ltd., Tokyo, Japan

[6]Japan Drug Development and Medical Affairs, Eli Lilly Japan K.K., Kobe, Japan

*AUTHOR FOR CORRESPONDENCE

Yoshinori Tanizawa

Japan Drug Development and Medical Affairs

Eli Lilly Japan K.K.

5-1-28, Isogamidori, Chuo-ku

Kobe-shi, Hyogo, 651-0086, Japan

E-mail: tanizawa_yoshinori@lilly.com

LIST OF TABLES AND FIGURES

Table S1 Demographics and clinical characteristics by HIT-6 score subgroup

Table S2 Demographics and clinical characteristics by MIBS-4 score subgroup

Table S3 Economic burden of migraine by HIT-6 score subgroup

Table S4 Economic burden of migraine by MIBS-4 score subgroup

Figure S1 Participant flow diagram

Figure S2 Headache symptoms and allodynia in the overall cohort and in the HIT-6 score subgroups based on level of impact (Severe, Substantial, Moderate, Little-to-none)

Figure S3 Headache symptoms and allodynia in the overall cohort and in the MIBS-4 score subgroups based on level of impact (Severe, Moderate, Mild, None)

Figure S4 **A**) MIDAS total score. **B**) Frequency of concern about headaches during the interictal period. **C**) MSQ score. **D**) WPAI-M score among all participants (Activity Impairment) or participants who are employed (overall *n* = 11,891 for Presenteeism and Work Productivity Loss; overall *n* = 11,981 for Absenteeism) in the overall cohort and in the HIT-6 score subgroups based on level of impact (Severe, Substantial, Moderate, Little-to-none)

Figure S5 Distribution of **A**) MIDAS grade and **B**) IMPAC grade (among participants with partners/spouses and/or children under 8 years of age; overall *n* = 12,775) in the overall cohort and in the HIT-6 score subgroups based on level of impact (Severe, Substantial, Moderate, Little-to-none)

Figure S6 Distribution of **A**) MIDAS grade and **B**) IMPAC grade (among participants with partners/spouses and/or children under 8 years of age; overall *n* = 12,775) in the overall cohort and in the MIBS-4 score subgroups based on level of impact (Severe, Moderate, Mild, None)

Figure S7 **A**) MIDAS total score. **B**) Frequency of concern about headaches during the interictal period. **C**) MSQ score. **D**) WPAI-M score among all participants (Activity Impairment) or participants who are employed (overall *n* = 11,891 for Presenteeism and Work Productivity Loss; overall *n* = 11,981 for Absenteeism) in the overall cohort and in the MIBS-4 score subgroups based on level of impact (Severe, Moderate, Mild, None)

Figure S8 **A**) Experience with medical treatment and diagnosis. **B**) Experience with drugs. **C**) mTOQ-6 score (in participants who had used OTC and/or prescription acute treatment for headache within the past year; overall *n* = 17,094) in the overall cohort and in the HIT-6 score subgroups based on level of impact (Severe, Substantial, Moderate, Little-to-none)

Figure S9 **A**) Experience with medical treatment and diagnosis. **B**) Experience with drugs. **C**) mTOQ-6 score (in participants who had used OTC and/or prescription acute treatment for headache within the past year; overall *n* = 17,094) in the overall cohort and in the MIBS-4 score subgroups based on level of impact (Severe, Moderate, Mild, None)

Figure S10 Benefits of preventive drug use among participants who did not select "I am not currently using headache prophylactic drugs" in the survey (overall *n* = 2628)

Table S1 Demographics and clinical characteristics by HIT-6 score subgroup

Variable	Overall[a] (*N* = 19,590)	Severe (*N* = 11,107)	Substantial (*N* = 3283)	Moderate (*N* = 3196)	Little-to-none (*N* = 2004)
MIBS-4 score, mean (SD)	3.2 (3.6)	4.4 (3.8)	2.5 (3.0)	1.5 (2.4)	0.8 (1.9)
HIT-6 score, mean (SD)	59.7 (7.8)	65.0 (4.7)	57.6 (1.1)	52.7 (1.7)	45.3 (3.4)
Age, years, mean (SD)	40.5 (13.1)	39.1 (12.3)	40.9 (13.3)	42.9 (14.1)	44.1 (14.7)
Female sex, *n* (%)	13,486 (68.8)	7965 (71.7)	2229 (67.9)	2121 (66.4)	1171 (58.4)
Employment, *n* (%)					
Full-time employee	7769 (39.7)	4380 (39.4)	1402 (42.7)	1231 (38.5)	756 (37.7)
Part-time, temporary, or contract employee	4955 (25.3)	2822 (25.4)	811 (24.7)	809 (25.3)	513 (25.6)
Self-employed	877 (4.5)	506 (4.6)	131 (4.0)	154 (4.8)	86 (4.3)
Stay-at-home spouse	2304 (11.8)	1289 (11.6)	378 (11.5)	405 (12.7)	232 (11.6)
Student with part-time job	699 (3.6)	407 (3.7)	118 (3.6)	99 (3.1)	75 (3.7)
Student with no part-time job	349 (1.8)	208 (1.9)	51 (1.6)	61 (1.9)	29 (1.4)
On long- or short-term leave	140 (0.7)	88 (0.8)	21 (0.6)	24 (0.8)	7 (0.3)
Unemployed, seeking employment	702 (3.6)	421 (3.8)	111 (3.4)	105 (3.3)	65 (3.2)
Unemployed, not seeking employment	1288 (6.6)	685 (6.2)	192 (5.8)	237 (7.4)	174 (8.7)
Prefer not to answer	507 (2.6)	301 (2.7)	68 (2.1)	71 (2.2)	67 (3.3)
Marriage status, *n* (%)					
Single, never married	8273 (42.2)	4803 (43.2)	1397 (42.6)	1267 (39.6)	806 (40.2)
Married	8881 (45.3)	4894 (44.1)	1500 (45.7)	1541 (48.2)	946 (47.2)
Living with a partner	816 (4.2)	517 (4.7)	132 (4.0)	108 (3.4)	59 (2.9)
Divorced	1263 (6.4)	690 (6.2)	218 (6.6)	210 (6.6)	145 (7.2)
Widowed	215 (1.1)	116 (1.0)	19 (0.6)	45 (1.4)	35 (1.7)
Prefer not to answer	142 (0.7)	87 (0.8)	17 (0.5)	25 (0.8)	13 (0.6)
Cohabitation status,[b] *n* (%)					
Live alone	4264 (21.8)	2451 (22.1)	707 (21.5)	658 (20.6)	448 (22.4)
Spouse/partner	9083 (46.4)	5064 (45.6)	1535 (46.8)	1551 (48.5)	933 (46.6)
Children/stepchildren/ grandchildren	6333 (32.3)	3697 (33.3)	1042 (31.7)	992 (31.0)	602 (30.0)
Parents/parents-in-law	5666 (28.9)	3241 (29.2)	946 (28.8)	916 (28.7)	563 (28.1)
Other people	1975 (10.1)	1099 (9.9)	366 (11.1)	309 (9.7)	201 (10.0)

Disease history during past 12 months,[b] *n* (%)					
Dizziness	5769 (29.4)	3663 (33.0)	869 (26.5)	797 (24.9)	440 (22.0)
Sinusitis	2195 (11.2)	1379 (12.4)	362 (11.0)	289 (9.0)	165 (8.2)
Insomnia	4220 (21.5)	2854 (25.7)	611 (18.6)	475 (14.9)	280 (14.0)
Depression/depressive state	3118 (15.9)	2230 (20.1)	406 (12.4)	301 (9.4)	181 (9.0)
Anxiety neurosis/anxiety disorder/panic disorder	2317 (11.8)	1668 (15.0)	300 (9.1)	216 (6.8)	133 (6.6)
Constipation	5133 (26.2)	3197 (28.8)	775 (23.6)	742 (23.2)	419 (20.9)
Chronic lower back pain	3320 (16.9)	2073 (18.7)	506 (15.4)	444 (13.9)	297 (14.8)
Stiff shoulders	11,395 (58.2)	6681 (60.2)	1889 (57.5)	1788 (55.9)	1037 (51.7)

[a]Data (except disease history) for the overall population also reported in Danno et al (2025) Neurol Ther. 14:335–356

[b]More than one answer possible

Abbreviations: HIT-6, Headache Impact Test-6; MIBS-4, Migraine Interictal Burden Scale-4; SD, standard deviation

Table S2 Demographics and clinical characteristics by MIBS-4 score subgroup

Variable	Overall[a] (*N* = 19,590)	Severe (*N* = 6336)	Moderate (*N* = 2886)	Mild (*N* = 2152)	None (*N* = 8216)
MIBS-4 score, mean (SD)	3.2 (3.6)	7.9 (2.1)	3.7 (0.5)	1.5 (0.5)	0.0 (0.0)
HIT-6 score, mean (SD)	59.7 (7.8)	63.9 (6.4)	60.3 (6.3)	59.4 (6.7)	56.4 (7.9)
Age, years, mean (SD)	40.5 (13.1)	38.2 (12.3)	41.4 (13.2)	41.6 (13.4)	41.8 (13.4)
Female sex, *n* (%)	13,486 (68.8)	4019 (63.4)	2022 (70.1)	1595 (74.1)	5850 (71.2)
Employment, *n* (%)					
Full-time employee	7769 (39.7)	2830 (44.7)	1143 (39.6)	793 (36.8)	3003 (36.6)
Part-time, temporary, or contract employee	4955 (25.3)	1440 (22.7)	754 (26.1)	559 (26.0)	2202 (26.8)
Self-employed	877 (4.5)	284 (4.5)	119 (4.1)	77 (3.6)	397 (4.8)
Stay-at-home spouse	2304 (11.8)	528 (8.3)	365 (12.6)	316 (14.7)	1095 (13.3)
Student with part-time job	699 (3.6)	271 (4.3)	91 (3.2)	83 (3.9)	254 (3.1)
Student with no part-time job	349 (1.8)	140 (2.2)	37 (1.3)	45 (2.1)	127 (1.5)
On long- or short-term leave	140 (0.7)	48 (0.8)	25 (0.9)	13 (0.6)	54 (0.7)
Unemployed, seeking employment	702 (3.6)	230 (3.6)	98 (3.4)	86 (4.0)	288 (3.5)
Unemployed, not seeking employment	1288 (6.6)	358 (5.7)	193 (6.7)	140 (6.5)	597 (7.3)
Prefer not to answer	507 (2.6)	207 (3.3)	61 (2.1)	40 (1.9)	199 (2.4)
Marriage status, *n* (%)					
Single, never married	8273 (42.2)	2978 (47.0)	1171 (40.6)	879 (40.8)	3245 (39.5)
Married	8881 (45.3)	2589 (40.9)	1356 (47.0)	1001 (46.5)	3935 (47.9)
Living with a partner	816 (4.2)	296 (4.7)	128 (4.4)	92 (4.3)	300 (3.7)
Divorced	1263 (6.4)	368 (5.8)	176 (6.1)	134 (6.2)	585 (7.1)
Widowed	215 (1.1)	45 (0.7)	39 (1.4)	26 (1.2)	105 (1.3)
Prefer not to answer	142 (0.7)	60 (0.9)	16 (0.6)	20 (0.9)	46 (0.6)
Cohabitation status,[b] *n* (%)					
Live alone	4264 (21.8)	1517 (23.9)	618 (21.4)	464 (21.6)	1665 (20.3)
Spouse/partner	9083 (46.4)	2663 (42.0)	1392 (48.2)	1036 (48.1)	3992 (48.6)
Children/stepchildren/ grandchildren	6333 (32.3)	1944 (30.7)	951 (33.0)	711 (33.0)	2727 (33.2)
Parents/parents-in-law	5666 (28.9)	1967 (31.0)	796 (27.6)	594 (27.6)	2309 (28.1)

Other people	1975 (10.1)	657 (10.4)	284 (9.8)	196 (9.1)	838 (10.2)
Disease history during past 12 months,[b] *n* (%)					
Dizziness	5769 (29.4)	2169 (34.2)	818 (28.3)	629 (29.2)	2153 (26.2)
Sinusitis	2195 (11.2)	888 (14.0)	308 (10.7)	216 (10.0)	783 (9.5)
Insomnia	4220 (21.5)	1850 (29.2)	606 (21.0)	421 (19.6)	1343 (16.3)
Depression/depressive state	3118 (15.9)	1490 (23.5)	433 (15.0)	262 (12.2)	933 (11.4)
Anxiety neurosis/anxiety disorder/panic disorder	2317 (11.8)	1134 (17.9)	338 (11.7)	208 (9.7)	637 (7.8)
Constipation	5133 (26.2)	1837 (29.0)	721 (25.0)	553 (25.7)	2022 (24.6)
Chronic lower back pain	3320 (16.9)	1141 (18.0)	528 (18.3)	361 (16.8)	1290 (15.7)
Stiff shoulders	11,395 (58.2)	3627 (57.2)	1713 (59.4)	1326 (61.6)	4729 (57.6)

[a]Data (except disease history) for the overall population also reported in Danno et al (2025) Neurol Ther. 14:335–356
[b]More than one answer possible
Abbreviations: HIT-6, Headache Impact Test-6; MIBS-4, Migraine Interictal Burden Scale-4; SD, standard deviation

Table S3 Economic burden of migraine by HIT-6 score subgroup

**Expense, JPY/Month, Mean (95% CI)**	**Overall (*N* = 19,590)**	**Severe (*N* = 11,107)**	**Substantial (*N* = 3283)**	**Moderate (*N* = 3196)**	**Little-to-none (*N* = 2004)**
Consultations and medications prescribed by the doctor	936.5 (874.0–999.0)	1295.1 (1189.0–1401.3)	618.9 (550.5–687.2)	425.7 (368.9–482.4)	283.6 (226.6–340.6)
Transportation to and from medical institutions	219.6 (190.6–248.5)	307.0 (259.3–354.8)	152.7 (102.9–202.4)	91.4 (56.3–126.5)	48.9 (34.3–63.5)
OTC headache drugs	794.3 (727.9–860.7)	939.0 (887.2–990.7)	814.8 (509.8–1119.7)	487.0 (458.4–515.7)	448.7 (153.8–743.7)
Supplements, food, etc.	465.0 (385.9–544.0)	633.3 (500.8–765.8)	372.5 (247.2–497.8)	189.0 (123.5–254.5)	123.5 (54.5–192.4)
Massage, physical therapy, going to a gym, etc.	477.4 (434.1–520.7)	604.7 (546.2–663.2)	424.1 (327.5–520.7)	295.1 (160.9–429.2)	150.0 (95.3–204.8)
Items for headache (pillows, fragrances, sunglasses, etc.)	135.7 (114.0–157.4)	197.7 (160.4–234.9)	83.8 (60.4–107.2)	44.7 (30.7–58.7)	22.6 (8.2–36.9)
Eating out, take-out (prepared foods, boxed lunches), and catering (delivery) for myself and my family during headaches	465.1 (419.2–510.9)	703.6 (625.2–782.0)	237.9 (188.1–287.7)	134.1 (94.8–173.4)	43.1 (21.6–64.7)
Taxi fares during headaches	113.2 (76.1–150.3)	179.1 (114.0–244.1)	36.5 (21.7–51.4)	24.9 (11.3–38.5)	14.4 (–1.7, 30.5)
Transportation of children during my headaches	35.3 (21.3–49.3)	56.9 (32.4–81.4)	8.7 (3.9–13.4)	8.6 (0.5–16.8)	1.7 (–0.6, 3.9)
Babysitting at home or elsewhere during a headache	54.5 (29.7–79.2)	88.0 (44.5–131.5)	18.4 (6.1–30.7)	7.6 (–0.1, 15.3)	2.2 (–0.2, 4.6)
Gifts of my gratitude to people for their help	83.2 (61.2–105.1)	128.5 (90.7–166.4)	36.6 (15.5–57.7)	13.9 (3.8–24.0)	18.4 (–3.7, 40.5)
Other	82.2 (29.7–134.6)	82.1 (25.7–138.5)	149.0 (–90.6, 388.5)	28.9 (1.7–56.2)	58.1 (–39.9, 156.1)
**Total**	**3861.7 (3634.4–4089.0)**	**5215.0 (4852.6–5577.3)**	**2953.7 (2477.9–3429.5)**	**1750.8 (1524.6–1977.0)**	**1215.1 (870.6–1559.7)**

Note: At the time of the study, 1000 JPY was approximately equivalent to US$7.00 and €6.40

Abbreviations: CI, confidence interval; HIT-6, Headache Impact Test-6; JPY, Japanese yen; OTC, over-the-counter

Table S4 Economic burden of migraine by MIBS-4 score subgroup

**Expense, JPY/Month, Mean (95% CI)**	**Overall (*N* = 19,590)**	**Severe (*N* = 6336)**	**Moderate (*N* = 2886)**	**Mild (*N* = 2152)**	**None (*N* = 8216)**
Consultations and medications prescribed by the doctor	936.5 (874.0–999.0)	1764.7 (1591.8–1937.6)	812.4 (683.7–941.2)	609.0 (539.0–679.1)	427.1 (386.1–468.2)
Transportation to and from medical institutions	219.6 (190.6–248.5)	458.8 (377.9–539.6)	203.8 (128.8–278.8)	124.0 (100.4–147.6)	65.6 (54.6–76.7)
OTC headache drugs	794.3 (727.9–860.7)	1176.6 (998.9–1354.2)	765.0 (705.8–824.1)	726.0 (675.7–776.3)	527.6 (452.6–602.6)
Supplements, food, etc.	465.0 (385.9–544.0)	865.1 (729.6–1000.6)	578.3 (159.2–997.4)	287.1 (174.5–399.7)	163.2 (118.2–208.1)
Massage, physical therapy, going to a gym, etc.	477.4 (434.1–520.7)	720.7 (635.5–805.9)	487.3 (382.4–592.1)	606.1 (378.9–833.3)	252.7 (215.1–290.2)
Items for headache (pillows, fragrances, sunglasses, etc.)	135.7 (114.0–157.4)	253.7 (211.2–296.2)	152.2 (47.3–257.0)	102.2 (66.4–138.0)	47.7 (35.6–59.8)
Eating out, take-out (prepared foods, boxed lunches), and catering (delivery) for myself and my family during headaches	465.1 (419.2–510.9)	850.6 (729.4–971.7)	391.2 (316.1–466.2)	418.4 (327.4–509.3)	206.0 (162.6–249.4)
Taxi fares during headaches	113.2 (76.1–150.3)	283.1 (170.3–395.9)	57.1 (32.3–81.8)	45.3 (13.7–76.9)	19.6 (10.0–29.3)
Transportation of children during my headaches	35.3 (21.3–49.3)	88.5 (46.8–130.2)	17.1 (1.0–33.1)	7.8 (–2.5, 18.0)	7.9 (1.7–14.1)
Babysitting at home or elsewhere during a headache	54.5 (29.7–79.2)	146.5 (71.3–221.7)	11.2 (0.8–21.5)	7.2 (–3.0, 17.4)	11.1 (0.9–21.2)
Gifts of my gratitude to people for their help	83.2 (61.2–105.1)	213.5 (147.1–279.8)	34.7 (18.3–51.1)	31.7 (10.7–52.7)	13.1 (6.1–20.1)
Other	82.2 (29.7–134.6)	155.4 (52.1–258.8)	28.0 (7.8–48.1)	12.1 (–1.0, 25.2)	63.1 (–33.0, 159.1)
**Total**	**3861.7 (3634.4–4089.0)**	**6977.1 (6379.4–7574.7)**	**3538.0 (3004.1–4071.9)**	**2976.9 (2618.2–3335.6)**	**1804.6 (1626.2–1983.1)**

Note: At the time of the study, 1000 JPY was approximately equivalent to US$7.00 and €6.40

Abbreviations: CI, confidence interval; JPY, Japanese yen; MIBS-4, Migraine Interictal Burden Scale-4; OTC, over-the-counter

Figure S1 Participant flow diagram. [a]Non-migraine group was not included in the current analysis

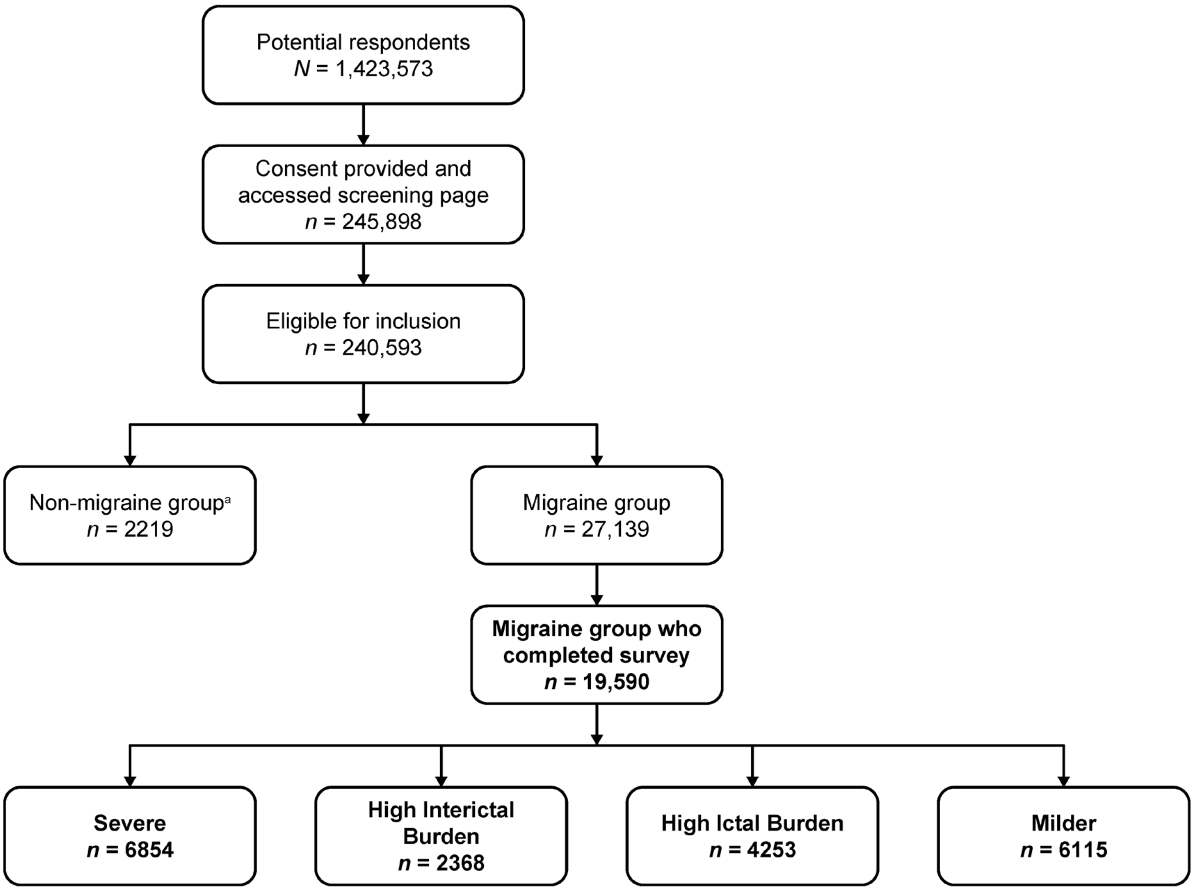

Figure S2 Headache symptoms and allodynia in the overall cohort and in the HIT-6 score subgroups based on level of impact (Severe, Substantial, Moderate, Little-to-none). **A**) MHD. **B**) Pain duration. As the distribution of pain duration was skewed, these data are presented as boxplots (the horizontal line within each box represents the median, the cross represents the mean, the bottom and top of each box represent the 25th and 75th percentile, respectively, and the error bars represent the range, excluding outliers [outside 1.5 times the interquartile range above the 75th percentile and below the 25th percentile]). **C**) Pain severity. **D**) ASC-12 scores. Abbreviations: ASC-12, Allodynia Symptoms Checklist-12; HIT-6, Headache Impact Test-6; MHD, monthly headache days; SD, standard deviation

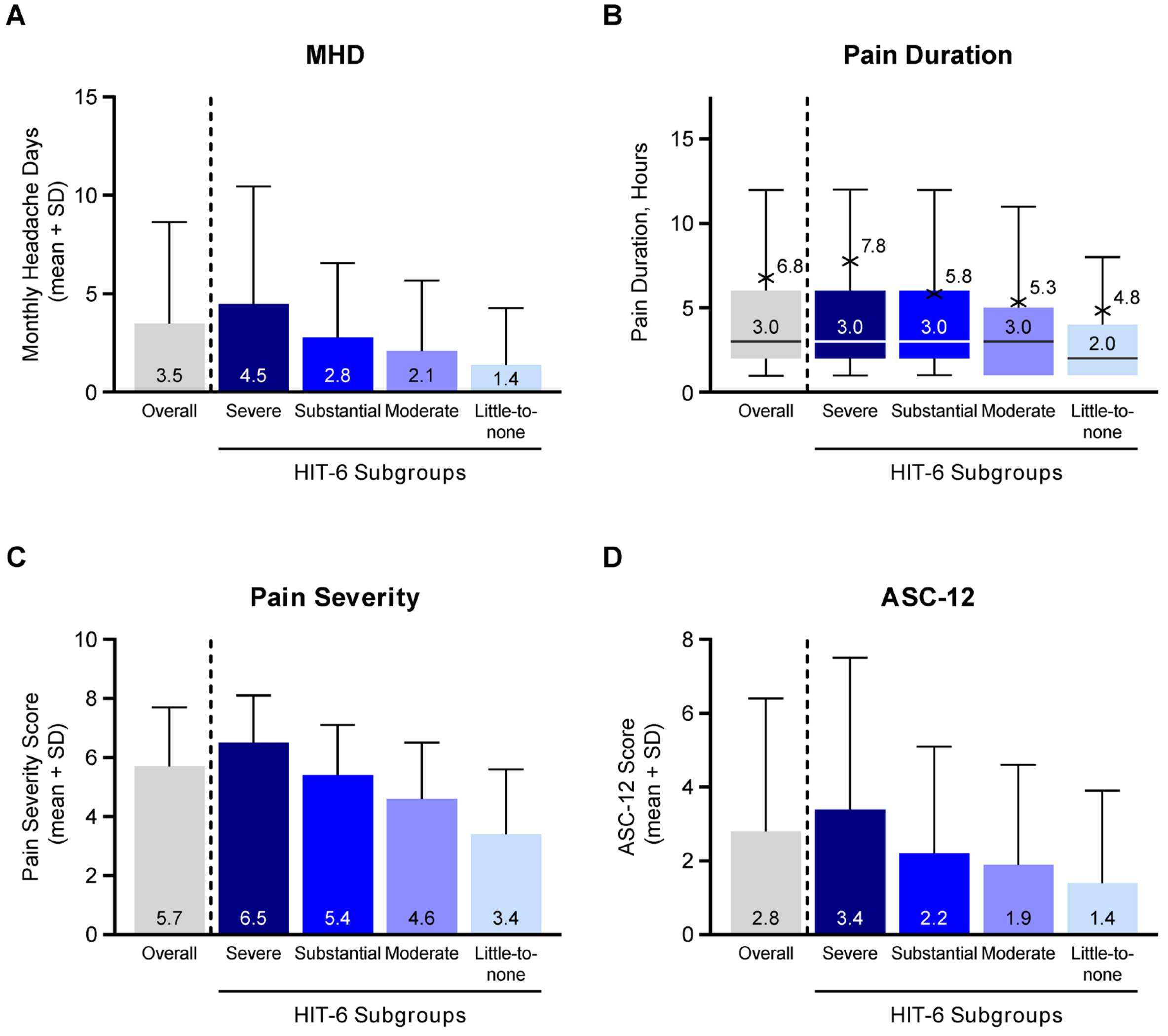

Figure S3 Headache symptoms and allodynia in the overall cohort and in the MIBS-4 score subgroups based on level of impact (Severe, Moderate, Mild, None). **A**) MHD. **B**) Pain duration. As the distribution of pain duration was skewed, these data are presented as boxplots (the horizontal line within each box represents the median, the cross represents the mean, the bottom and top of each box represent the 25th and 75th percentile, respectively, and the error bars represent the range, excluding outliers [outside 1.5 times the interquartile range above the 75th percentile and below the 25th percentile]). **C**) Pain severity. **D**) ASC-12 scores. Abbreviations: ASC-12, Allodynia Symptoms Checklist-12; MHD, monthly headache days; MIBS-4, Migraine Interictal Burden Scale-4; SD, standard deviation

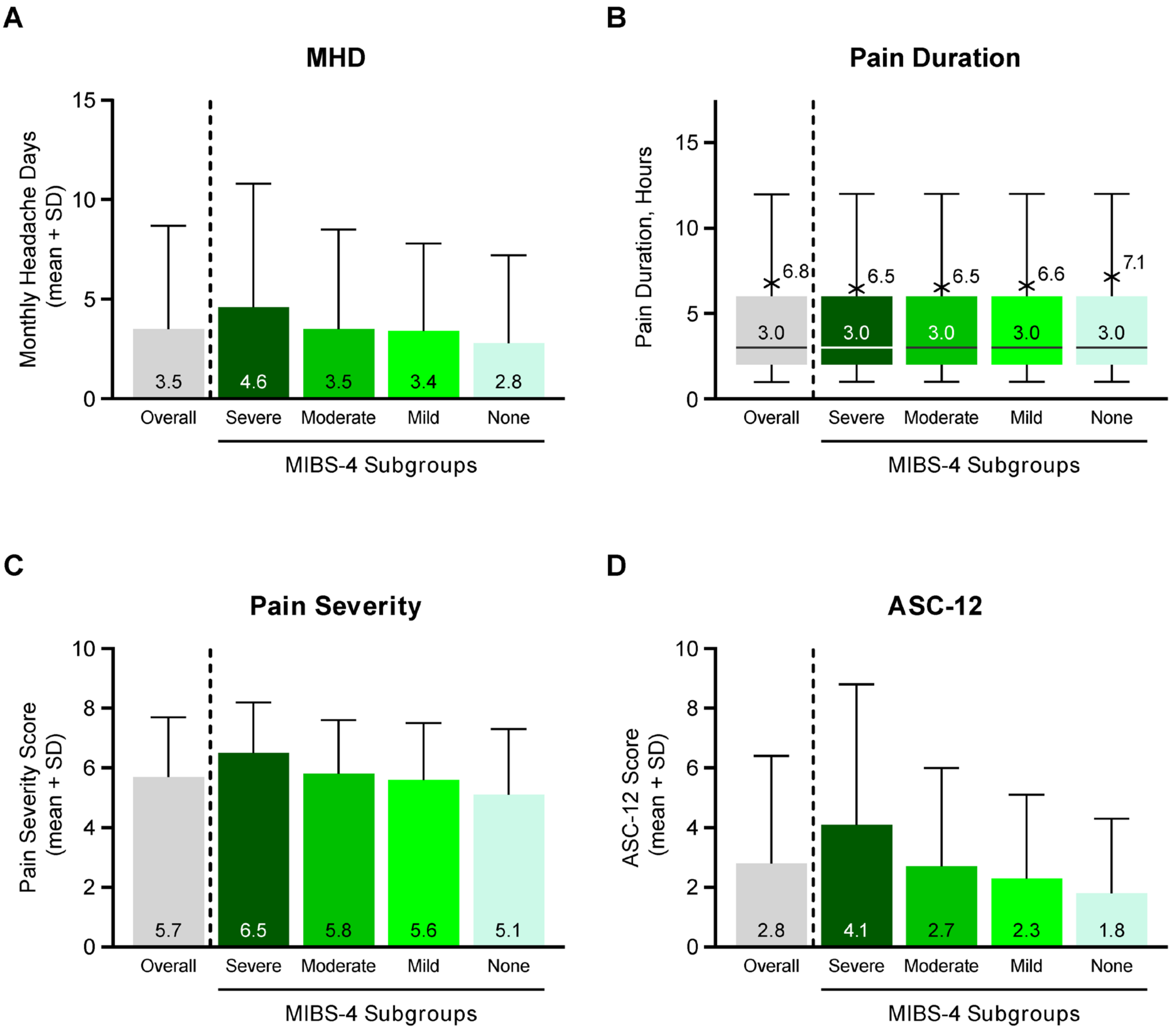

Figure S4 **A**) MIDAS total score. **B**) Frequency of concern about headaches during the interictal period. **C**) MSQ score. **D**) WPAI-M score among all participants (Activity Impairment) or participants who are employed (overall *n* = 11,891 for Presenteeism and Work Productivity Loss; overall *n* = 11,981 for Absenteeism) in the overall cohort and in the HIT-6 score subgroups based on level of impact (Severe, Substantial, Moderate, Little-to-none). Abbreviations: HIT-6, Headache Impact Test-6; MIDAS, Migraine Disability Assessment; MSQ, Migraine-Specific Quality-of-Life Questionnaire; SD, standard deviation; WPAI-M, Work Productivity and Activity Impairment-Migraine

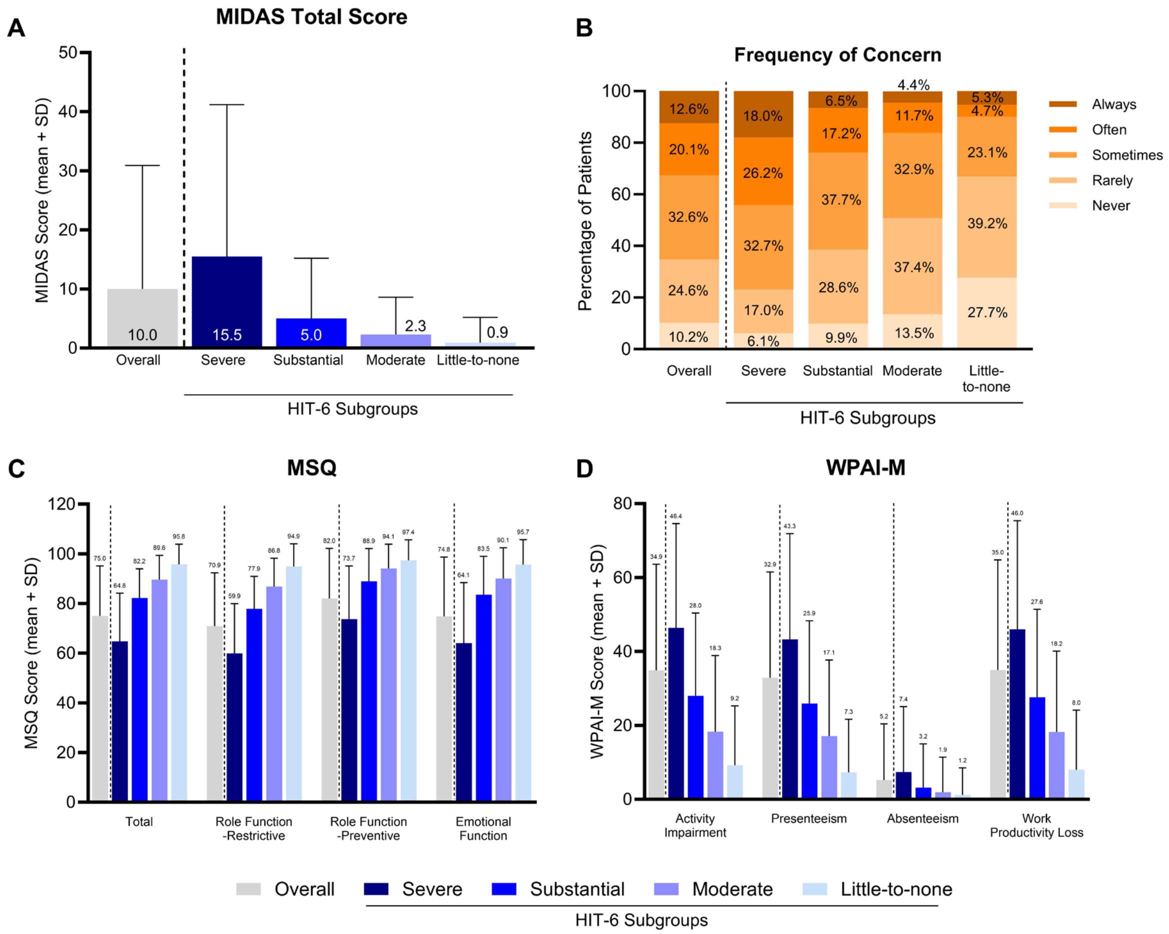

Figure S5 Distribution of **A**) MIDAS grade and **B**) IMPAC grade (among participants with partners/spouses and/or children under 8 years of age; overall *n* = 12,775) in the overall cohort and in the HIT-6 score subgroups based on level of impact (Severe, Substantial, Moderate, Little-to-none). Abbreviations: HIT-6, Headache Impact Test-6; IMPAC, Impact of Migraine on Partners and Adolescent Children Scale; MIDAS, Migraine Disability Assessment

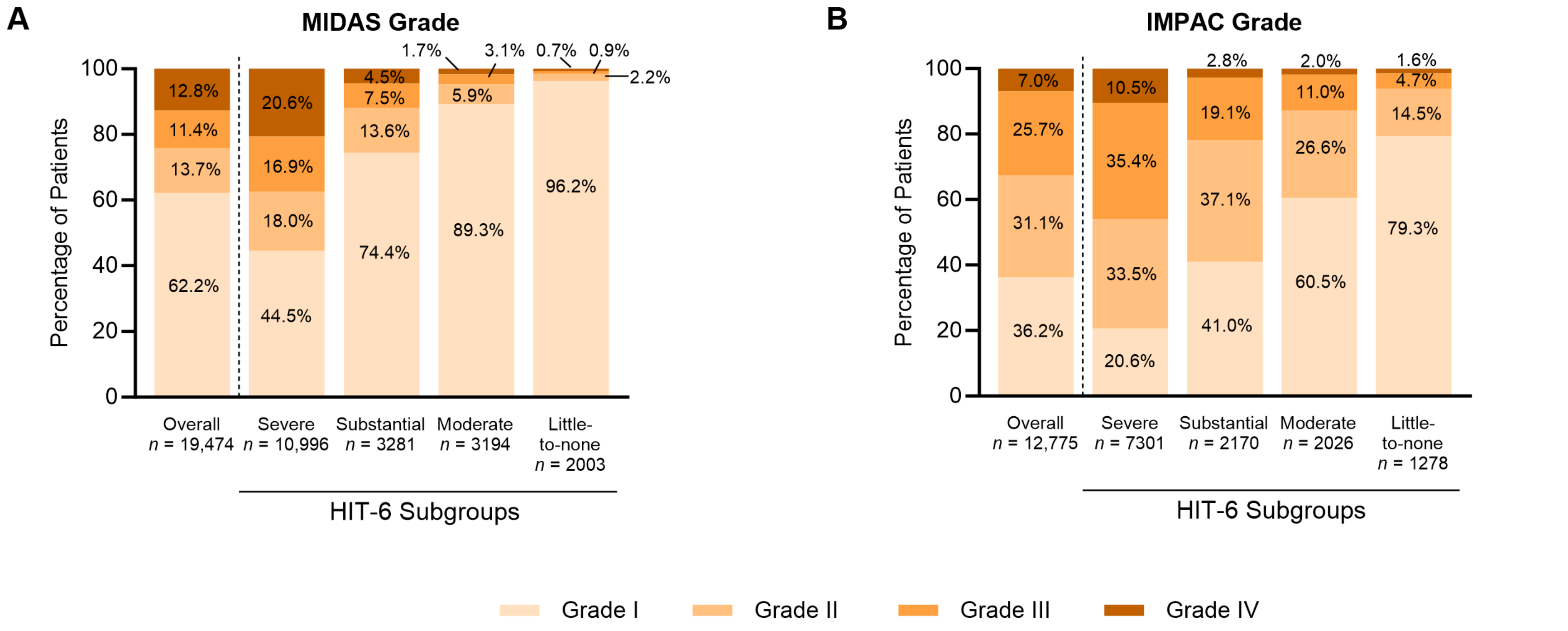

Figure S6 Distribution of **A**) MIDAS grade and **B**) IMPAC grade (among participants with partners/spouses and/or children under 8 years of age; overall *n* = 12,775) in the overall cohort and in the MIBS-4 score subgroups based on level of impact (Severe, Moderate, Mild, None). Abbreviations: IMPAC, Impact of Migraine on Partners and Adolescent Children Scale; MIBS-4, Migraine Interictal Burden Scale-4; MIDAS, Migraine Disability Assessment

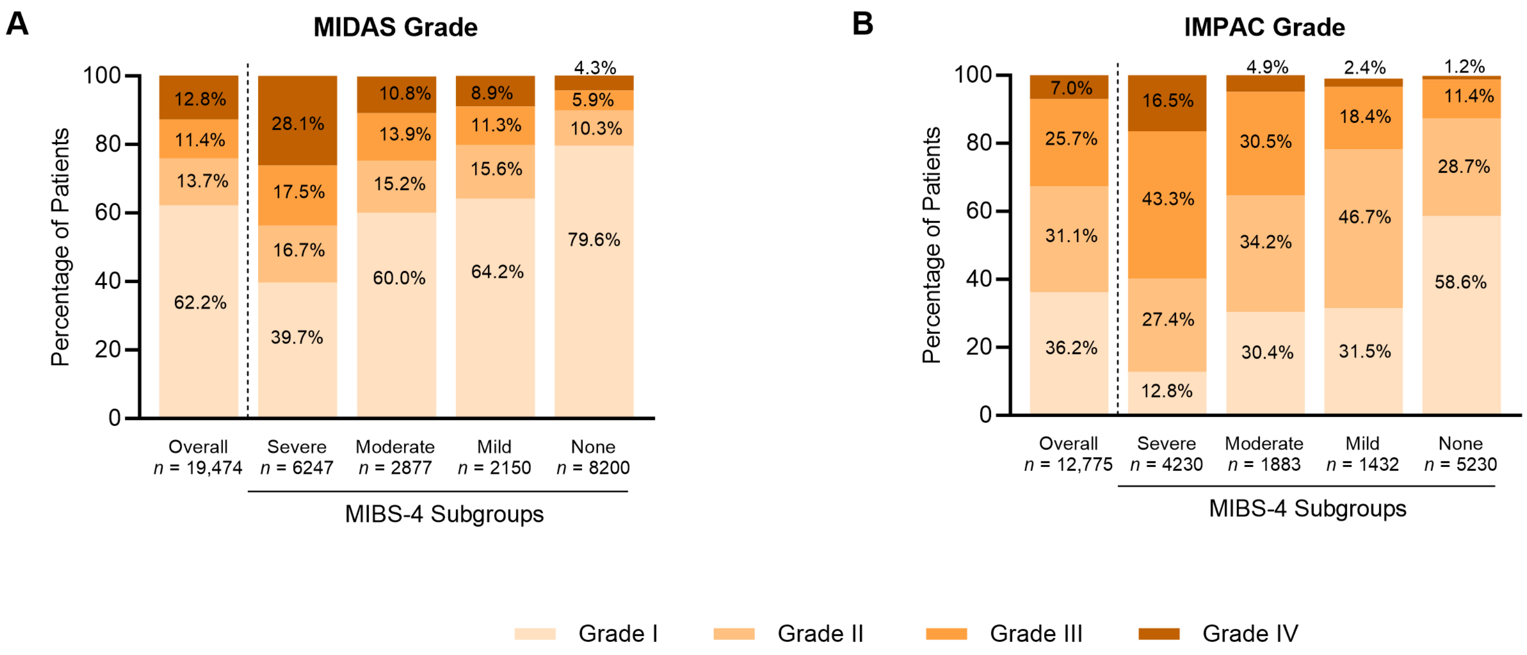

Figure S7 **A**) MIDAS total score. **B**) Frequency of concern about headaches during the interictal period. **C**) MSQ score. **D**) WPAI-M score among all participants (Activity Impairment) or participants who are employed (overall *n* = 11,891 for Presenteeism and Work Productivity Loss; overall *n* = 11,981 for Absenteeism) in the overall cohort and in the MIBS-4 score subgroups based on level of impact (Severe, Moderate, Mild, None). Abbreviations: MIBS-4, Migraine Interictal Burden Scale-4; MIDAS, Migraine Disability Assessment; MSQ, Migraine-Specific Quality-of-Life Questionnaire; SD, standard deviation; WPAI-M, Work Productivity and Activity Impairment-Migraine

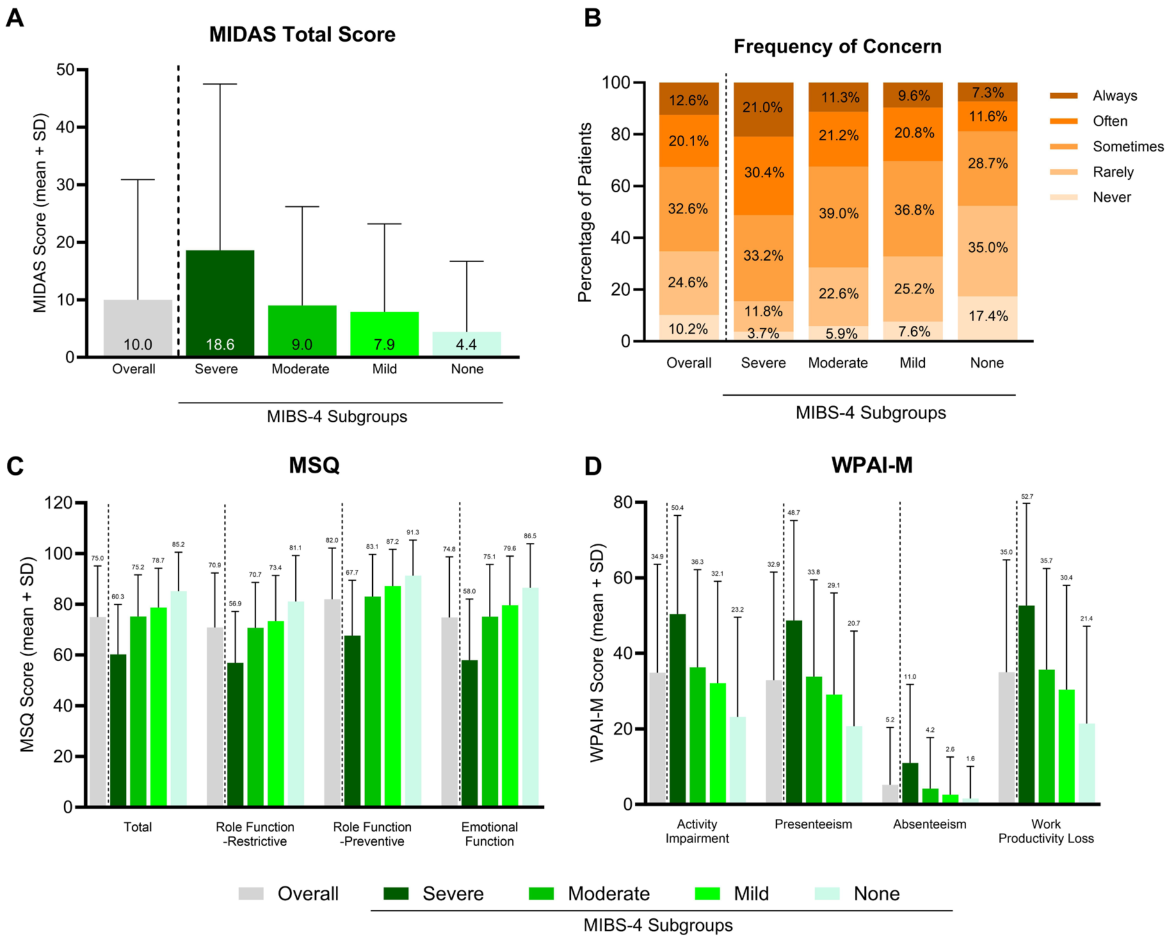

Figure S8 **A**) Experience with medical treatment and diagnosis. **B**) Experience with drugs. **C**) mTOQ-6 score (in participants who had used OTC and/or prescription acute treatment for headache within the past year; overall *n* = 17,094) in the overall cohort and in the HIT-6 score subgroups based on level of impact (Severe, Substantial, Moderate, Little-to-none). Abbreviations: CGRP, calcitonin gene-related peptide; HIT-6, Headache Impact Test-6; mAbs, monoclonal antibodies; mTOQ-6, Migraine Treatment Optimization Questionnaire-6; OTC, over-the-counter; SD, standard deviation

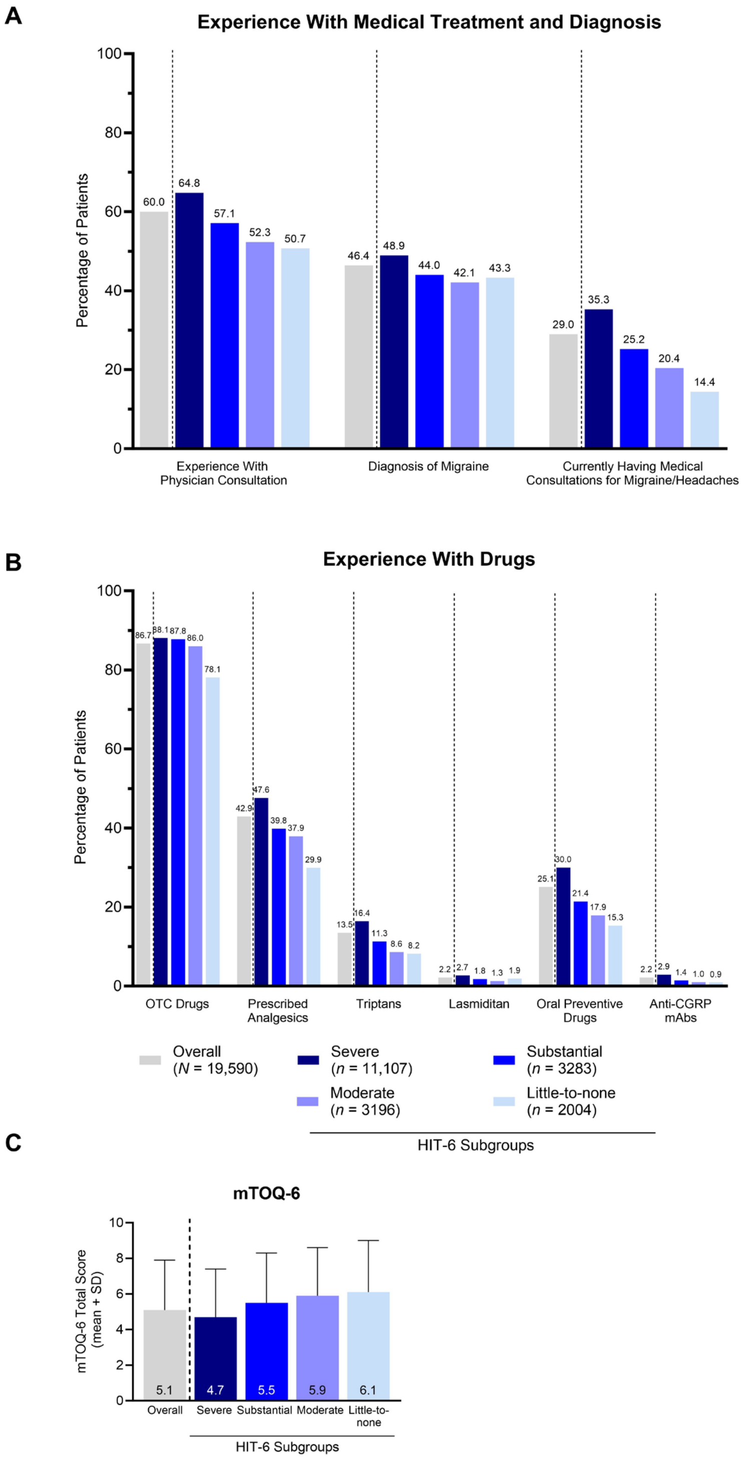

Figure S9 **A**) Experience with medical treatment and diagnosis. **B**) Experience with drugs. **C**) mTOQ-6 score (in participants who had used OTC and/or prescription acute treatment for headache within the past year; overall *n* = 17,094) in the overall cohort and in the MIBS-4 score subgroups based on level of impact (Severe, Moderate, Mild, None). Abbreviations: CGRP, calcitonin gene-related peptide; mAbs, monoclonal antibodies; MIBS-4, Migraine Interictal Burden Scale-4; mTOQ-6, Migraine Treatment Optimization Questionnaire-6; OTC, over-the-counter; SD, standard deviation

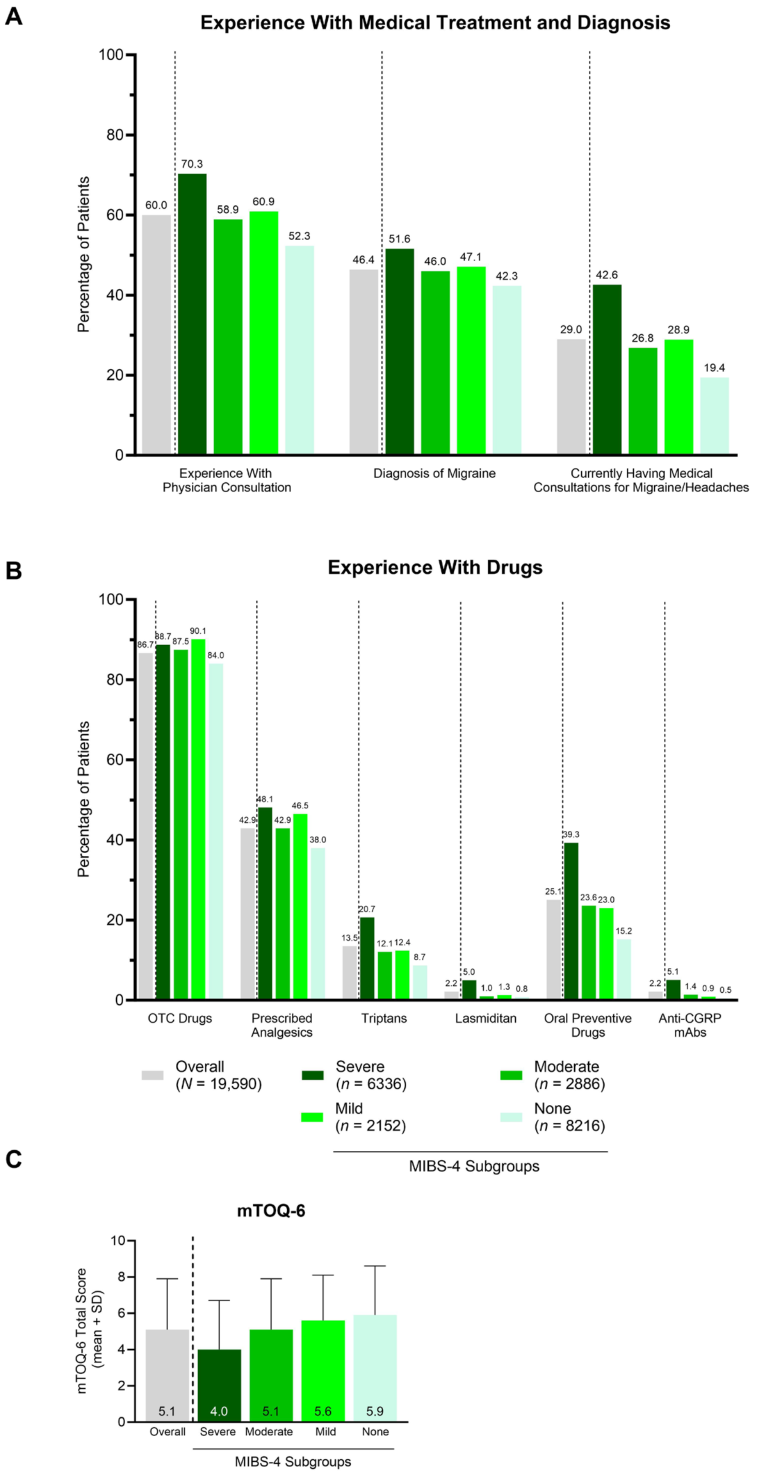

Figure S10 Benefits of preventive drug use among participants who did not select "I am not currently using headache prophylactic drugs" in the survey (overall n = 2628). HIT-High subgroups are shown in blue, and HIT-Low subgroups are shown in green. MIBS-High subgroups are shown in dark colors, and MIBS-Low subgroups are shown in light colors. Pairwise comparisons between subgroups were analyzed by chi-square tests. *p<0.05, **p<0.01. Abbreviations: HIT, Headache Impact Test; MIBS, Migraine Interictal Burden Scale

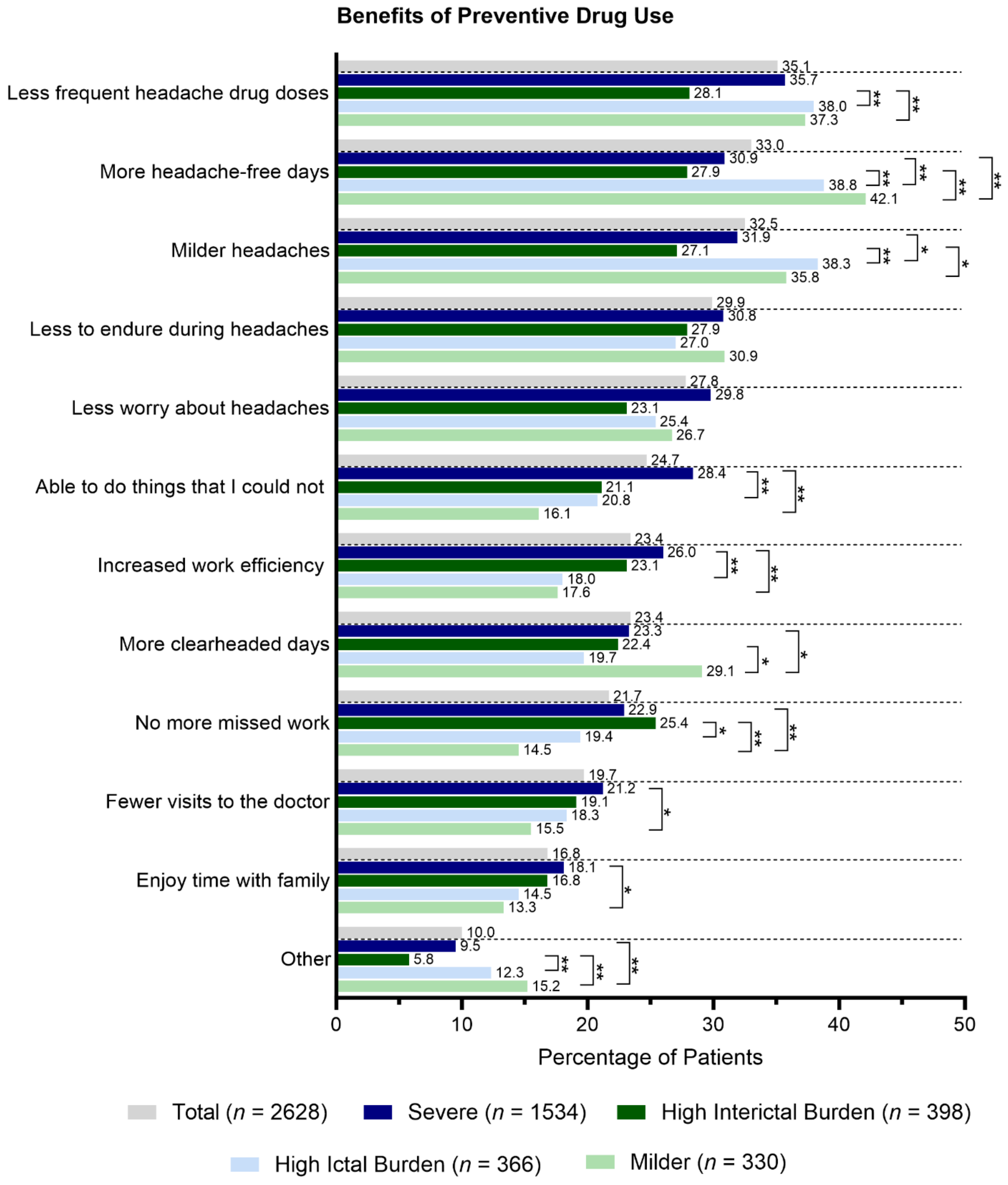

Supplement: Supplementary file 1 — Supplementary Material 1. [file 10194_2025_2079_MOESM1_ESM.pdf]
